# The Chromatin Remodelling Enzymes SNF2H and SNF2L Position Nucleosomes adjacent to CTCF and Other Transcription Factors

**DOI:** 10.1371/journal.pgen.1005940

**Published:** 2016-03-28

**Authors:** Nicola Wiechens, Vijender Singh, Triantaffyllos Gkikopoulos, Pieta Schofield, Sonia Rocha, Tom Owen-Hughes

**Affiliations:** 1 Centre for Gene Regulation and Expression, College of Life Sciences, University of Dundee, Dundee, United Kingdom; 2 Division of Biological Chemistry and Drug Discovery, College of Life Sciences, University of Dundee, Dundee, United Kingdom; Babraham Institute, UNITED KINGDOM

## Abstract

Within the genomes of metazoans, nucleosomes are highly organised adjacent to the binding sites for a subset of transcription factors. Here we have sought to investigate which chromatin remodelling enzymes are responsible for this. We find that the ATP-dependent chromatin remodelling enzyme SNF2H plays a major role organising arrays of nucleosomes adjacent to the binding sites for the architectural transcription factor CTCF sites and acts to promote CTCF binding. At many other factor binding sites SNF2H and the related enzyme SNF2L contribute to nucleosome organisation. The action of SNF2H at CTCF sites is functionally important as depletion of CTCF or SNF2H affects transcription of a common group of genes. This suggests that chromatin remodelling ATPase’s most closely related to the Drosophila ISWI protein contribute to the function of many human gene regulatory elements.

## Author Summary

CTCF is a transcriptional regulator acting as an insulator element interfering with enhancer function and as a boundary between chromatin domains. CTCF has been shown to organise an exquisite array of phased nucleosomes flanking its binding sites. Here we identified SNF2H as the enzyme primarily responsible for organising the extended arrays of nucleosomes adjacent to CTCF sites. We find that SNF2H acts to maintain the occupancy of CTCF at its binding sites, but does not act as a general loading factor for CTCF’s binding partner cohesin. SNF2H’s action at CTCF sites is functionally important as overlapping cohorts of genes are affected by depletion of CTCF or SNF2H. Other transcription factors also organise nucleosomes and we find that the SNF2H and the related enzyme SNF2L contribute to organising nucleosomes at many of these sites.

## Introduction

The genomes of eukaryotes exist predominantly as chromatin. The fundamental subunit of chromatin is the nucleosome which consists of 147 bp of DNA wrapped around an octamer of histone proteins [1]. Typically nucleosomes are distributed along DNA with defined spacing at distinct loci in a given cell type [2]. In addition, nucleosomes exhibit distinct translational positioning with respect to certain genomic features such as promoters [3–5], origins of DNA replication [6, 7] and the binding sites for transcription factors such as CTCF [8, 9].

CTCF binding has also been found to play a key contribution to the function of insulator elements [10]. Insulators are genetic elements that act to limit the range over which enhancers can act to regulate a gene [11]. Sites occupied by CTCF are frequently observed to also be enriched for subunits of the cohesin complex [12]. Cohesin is a multi- protein complex consisting of two SMC proteins (SMC1 and SMC3) and Rad21 (Scc1) and STAG (Scc3). It forms a ring like complex capable of encircling two DNA strands [13]. This function for cohesin was originally characterised as playing a key role in the association of newly replicated sister chromatids until they segregate in anaphase. However, subsequently it has become clear that cohesin can also mediate interactions between chromosomal loci during interphase. For example, interactions between cohesin and mediator have been found to mediate looping interactions between promoters and enhancers [14]. The combined action of both CTCF and cohesin mediates long range interactions and effects on gene expression [15–18]. In addition, recruitment of cohesin to CTCF binding sites also contributes to insulator activity [19–21]. However, in some cases CTCF sites remain functional following depletion of cohesin [18, 22].

ATP-dependent chromatin remodelling enzymes have been found to play an important role in establishing the positioning of many nucleosomes within the genomes of model organisms [23]. More recently several studies have addressed the roles of members of this family of ATPases in the human genome. For example the human ISWI related remodelling enzymes SNF2H (also known as SMARCA5) has been found to contribute to DNA repair [24], and in a partially redundant fashion to the organisation of a subset of DNase hypersensitive sites [25]. This study also found that SNF2H and CHD4 associate with a significant number of CTCF binding sites and a previous study demonstrated a role for the enzyme CHD8 at CTCF sites [26]. Both CHD8 and SNF2H have been shown to affect enhancer blocking mediated by CTCF at individual loci [26][27]. More recently, the bromodomain PHD finger-containing transcription factor (BPTF) subunit of the NURF complex has been observed to contribute to localised chromatin accessibility at CTCF sites and the regulation of CTCF target genes [28].

SNF2H is known to function as the catalytic ATPase in at least five distinct complexes in mammalian cells, namely ACF, CHRAC, WICH, RSF and NoRC [29]. The accessory subunits with which the SNF2H ATPase subunit is associated with varies in the different complexes. For example, SNF2H is found in association with WSTF in the WICH complex, with Tip5 in NoRC, Acf1 in ACF, and with both Acf1 and CHRAC 15/17 in CHRAC [29]. The related ATPase SNF2L is the ATPase subunit in the Cerf and NURF complexes [29].

To our knowledge no studies to date have investigated the contribution of different remodelling enzymes to the establishment of organised nucleosomal arrays adjacent to CTCF and other transcription factor binding sites. Here we find that SNF2H plays a major role in the establishment of ordered arrays of phased nucleosomes flanking CTCF binding sites. The related enzyme SNF2L plays a minor role at CTCF sites, and contributes to nucleosome positioning adjacent to other transcription factors. Depletion of SNF2H results in alterations to the expression of many CTCF dependent genes indicating a role for this enzyme in CTCF function and raising the possibility that nucleosome phasing contributes to function at gene regulatory elements.

## Results

### Chromatin organisation at promoters

**Fig 1:**
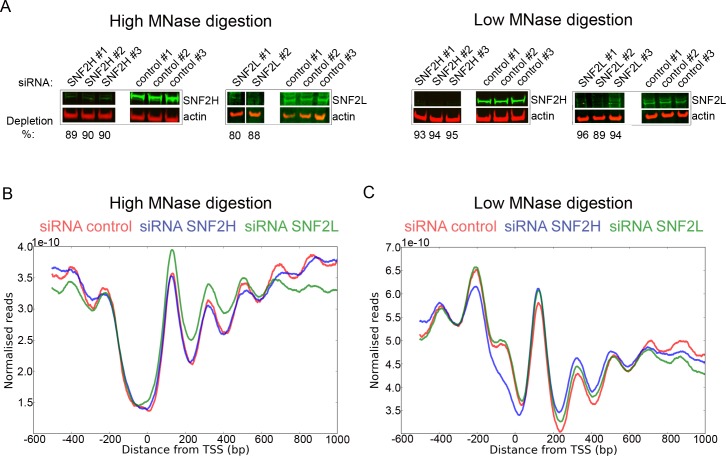
A siRNA depletion approach to study the roles of chromatin remodelling enzymes in nucleosome organisation. HeLa cells were depleted of SNF2H and SNF2L protein, respectively, and mono nucleosomes were obtained using high or low MNase digestion. (A) Western blot showing siRNA knock down of SNF2H and SNF2L proteins compared to control knock down using a scramble oligo for high (left panels) and low (right panels) MNase digestion samples. Whole cells extracts made with NP40 (SNF2H) and urea sample buffer (SNF2L) were analysed using Western blotting. Level of depletion was determined using infrared fluorescence normalized to a beta-actin loading control. (B, C) Alignment of high depth mono nucleosome reads to ubiquitously promoters following depletion of SNF2H (blue), SNF2L (green), and the control depletion (red) after high MNase digest (B, average fragment length of 147 bp) and low MNase digest (C, average fragment length of 169 bp).

To investigate the contributions of ATP-dependent chromatin remodelling enzymes in nucleosome organisation, we adopted an siRNA based approach to deplete selected enzymes in cultured HeLa cells. CHD1, CHD2, CHD4 (mi-2), SNF2L and SHF2H could be depleted to between 80% and 96% as judged by western blotting (Fig 1A and S1 Fig). Chromatin isolated from these cells was digested with micrococcal nuclease and the nucleosomal ladder was assessed by gel electrophoresis. Subtle changes in the digestion pattern were observed, but in all cases a distinct species of approximately 150 bp was detected (S1 Fig). In order to characterise the distribution of these nucleosomal DNA fragments, they were subject to high throughput sequencing to a depth of 40–350 million paired reads per repeat.

We investigated how depletion of these enzymes affected the organisation of nucleosomes at the promoters of ubiquitously expressed genes. We noticed variation in distribution of nucleosomes across promoters between experimental repeats and realised that this pattern varied with the extent of MNase digestion (Fig 1B and 1C). The extent of MNase digestion could be assessed from the mean length of the mono nucleosome fragments. With fragments digested to a mean length of 147 bp the nucleosome free region was distinct (Fig 1B). With digestion to 169 bp the nucleosome depleted region is partially filled and the -1 nucleosome more prominent (Fig 1C). Using controls with comparable MNase digestion it was not possible to detect significant changes in nucleosome distribution following depletion of SNF2H, SNF2L (Fig 1B and 1C), CHD1, CHD2 or CHD4 (S1 Fig).

### SNF2H acts to organise nucleosomes at CTCF binding sites

**Fig 2:**
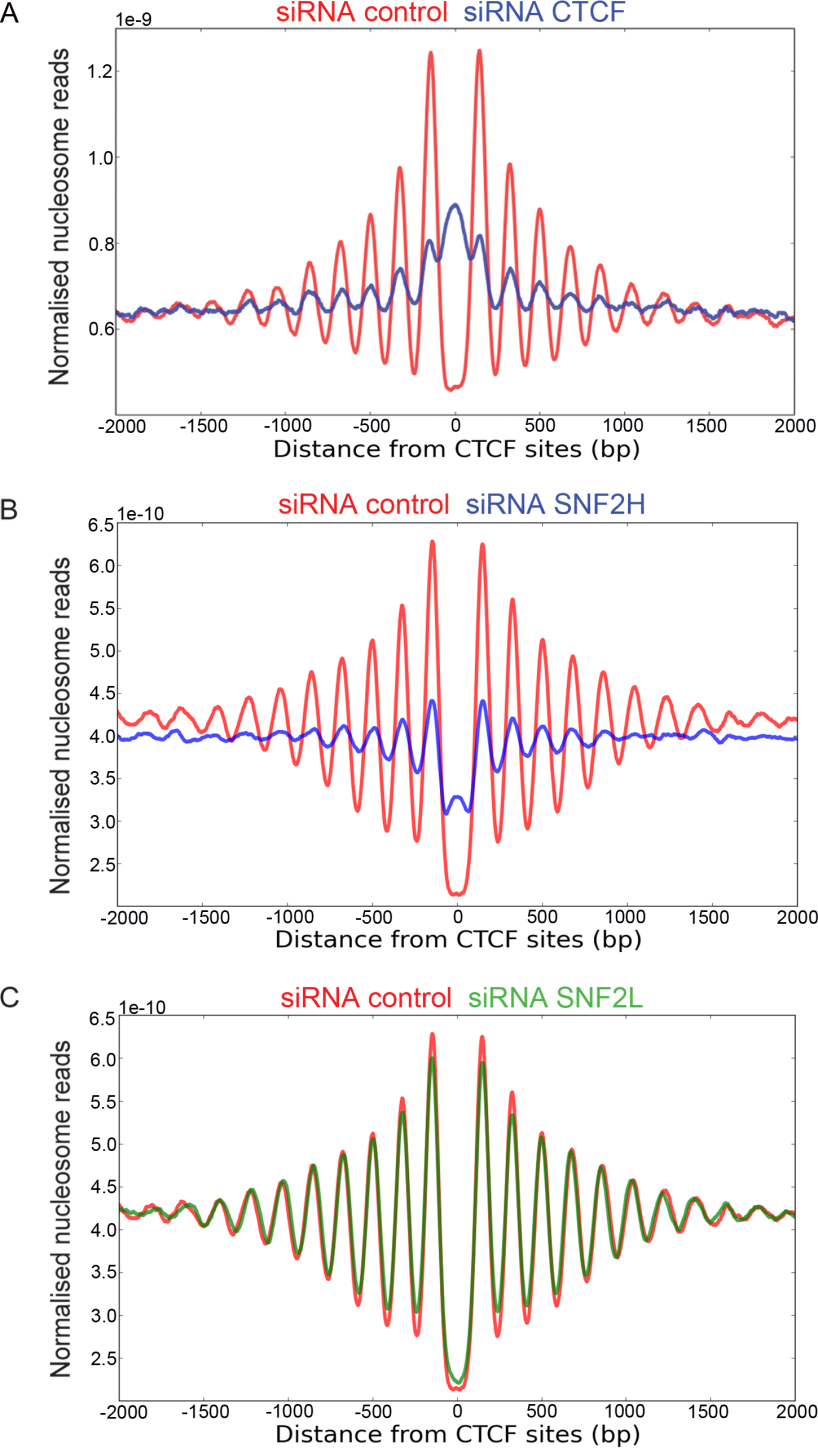
SNF2H organizes nucleosomes at CTCF binding sites. (A-C) Mono nucleosomes were isolated from cell lines following siRNA depletion of proteins indicated. Sequencing of the nucleosomal DNA enabled reads to be mapped with respect to CTCF sites. The strong nucleosome organization flanking the CTCF binding sites is disrupted following depletion of either CTCF (A) or SNF2H (B). The depletion of SNF2L (C) shows only a small effect. The same control depletion data is plotted in (B) and (C).

We next investigated the organisation of nucleosomes adjacent to CTCF binding sites where strikingly well organised arrays of around 20 positioned nucleosomes have been reported previously [8](Fig 2A). As expected, the organisation of nucleosomes is dependent on CTCF as siRNA depletion of CTCF reduces the nucleosomal pattern (Fig 2A). While depletion of CHD1, CHD2 and CHD4 had little effect on this pattern (S2 Fig), depletion of SNF2H resulted in a significant loss of nucleosome organisation at these sites (Fig 2B). Depletion of SNF2L had a small effect on the nucleosomes adjacent to the CTCF binding site, with progressively weaker effects at nucleosomes located further away (Fig 2C). As SNF2H is present within multiple distinct remodelling complexes in human cells, we next attempted to distinguish which complexes were involved. siRNA depletion of the ACF1, RSF1, TIP5 and WSTF subunits of these complexes did not disrupt nucleosome organisation to the same extent as observed for SNF2H (S3 Fig). We conclude that different SNF2H containing complexes may function with partial redundancy. SNF2L is known to form a complex with subunits of the human NURF complex including BPTF [30, 31]. Depletion of BPTF resulted in a change to the organisation of nucleosomes immediately adjacent to CTCF sites related to that observed with SNF2L suggesting that SNF2L functions at CTCF sites as a component of the NURF complex (S3 Fig).

### Interdependence of SNF2H, CTCF and cohesin occupancy

**Fig 3:**
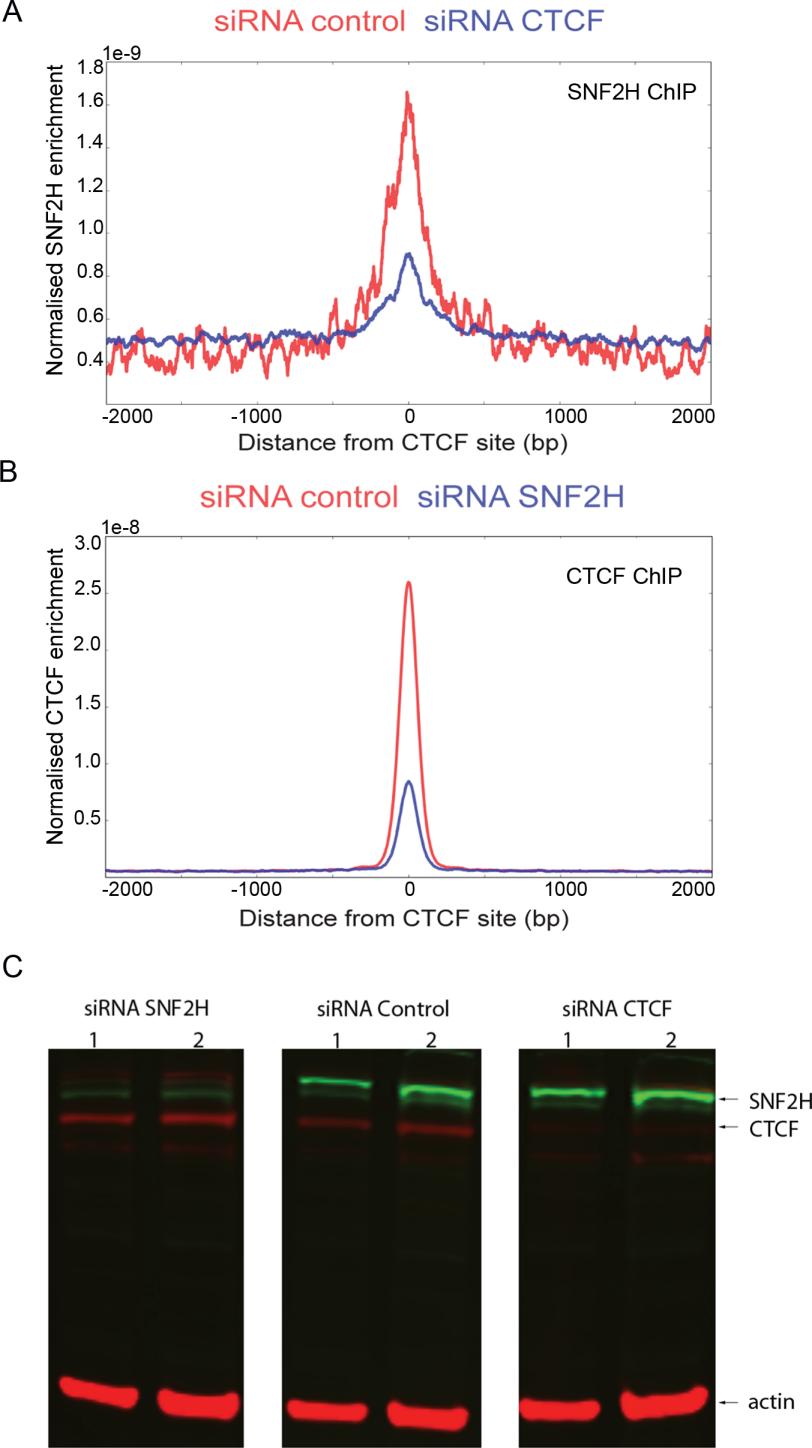
Interdependence of SNF2H and CTCF binding. (A) Chromatin of control cells (red) and CTCF depleted (blue) HeLa cells was immuno-precipitated using an anti-SNF2H antibody and the resulting DNA fragments sequenced. SNF2H is enriched at CTCF sites in comparison to the surrounding DNA, and this enrichment is reduced following depletion of CTCF. (B) ChIP seq experiment for CTCF showing enrichment at CTCF sites (red). Following depletion of SNF2H CTCF occupancy is reduced (blue). (C) Protein levels of SNF2H (green bands) and CTCF (top red bands) in cells depleted for SNF2H, control cells or CTCF depleted cells. Two different quantities (lane 1 and 2) of whole cell extracts of the respective cells were immuno-blotted for SNF2H, CTCF and beta-actin as a loading control. Depletion of SNF2H does not result in any global change in CTCF protein levels.

As SNF2H affects nucleosome organisation at CTCF sites, we examined whether SNF2H is physically associated with CTCF sites by ChIP. SNF2H is enriched at CTCF sites and enrichment at these sites is reduced following depletion of CTCF (Fig 3A). We also noticed that nucleosome occupancy increased at CTCF sites following depletion of SNF2H (Fig 2B). This led us to investigate whether CTCF occupancy was affected following depletion of SNF2H. The ChIP signal for CTCF was indeed found to be reduced following depletion of SNF2H (Fig 3B). This indicates that in addition to organising nucleosomes adjacent to CTCF, SNF2H acts to maintain high CTCF occupancy.

**Fig 4:**
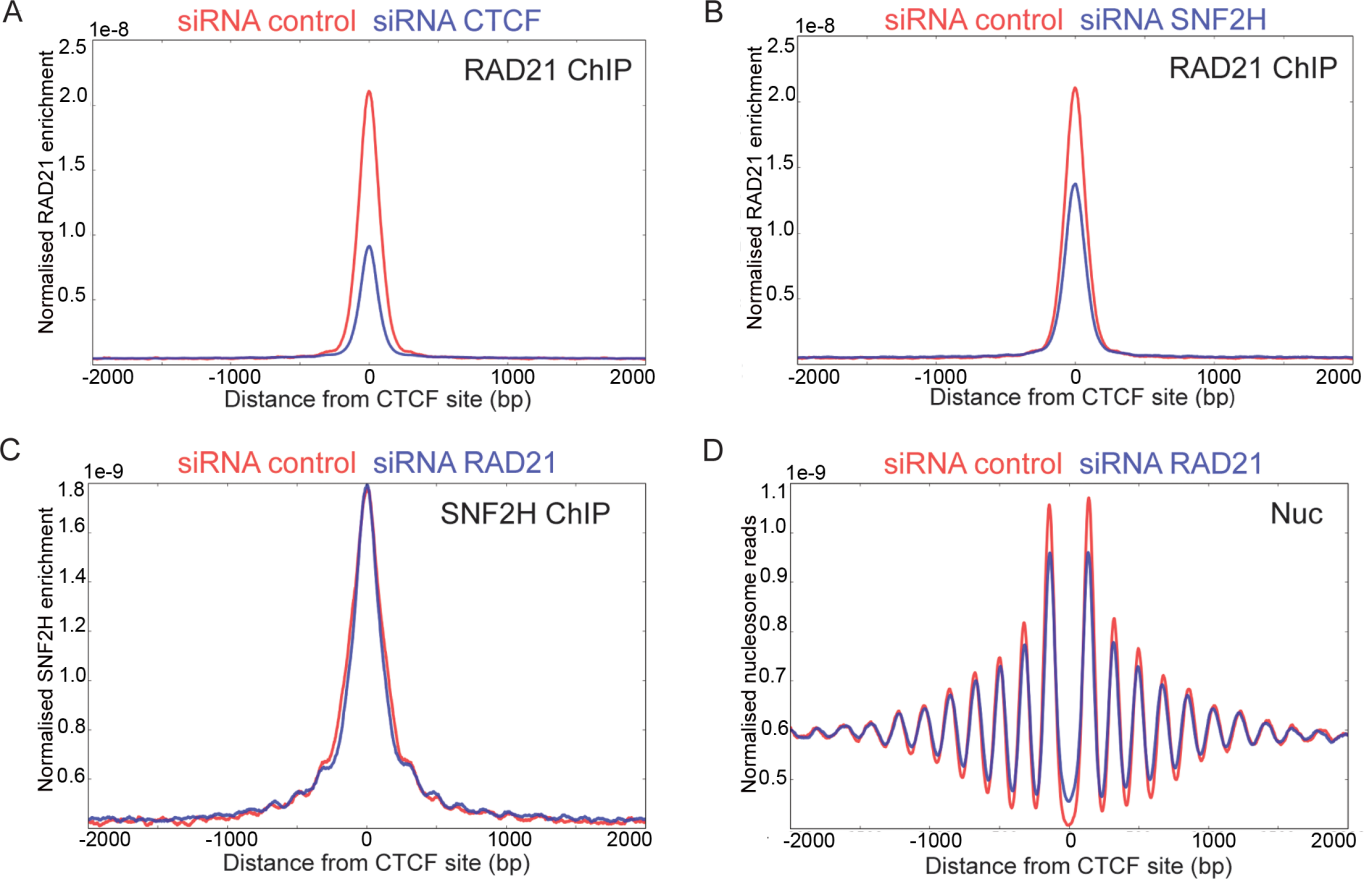
Cohesin does not organize nucleosomes at CTCF sites. (A, B) ChIP seq for RAD21 was plotted with respect to CTCF sites following depletion of CTCF (A) or SNF2H (B). In each case enrichment for RAD21 is reduced following depletion (blue) in comparison to control depletions (red). (C) ChIP seq showing SNF2H enrichment at CTCF sites in RAD21 (blue) depleted and control (red) cells. SNF2H enrichment is not affected by depletion of RAD21. (D) Depletion of RAD21 (blue) has little effect on nucleosome organization at CTCF sites in comparison to a control depletion.

Given that sites bound by CTCF are often also found to be enriched for cohesin, we investigated the effect of depleting CTCF or SNF2H on ChIP enrichment for the cohesin subunit Rad21. Fig 4A shows that enrichment for Rad21 is reduced by approximately 64% following depletion of CTCF. This is consistent with previous studies showing that recruitment of Rad21 to CTCF sites is dependent on CTCF [12, 19–21]. Enrichment of Rad21 is also reduced following depletion of SNF2H (Fig 4B). The reduction in occupancy (36%) is likely to be attributable to 68% reduction of CTCF occupancy following depletion of SNF2H rather than a direct role for SNF2H in Rad21 loading. We also observe that depletion of Rad21 had no effect on SNF2H recruitment to CTCF binding sites (Fig 4C) and consistent with this, depletion of Rad21 had little effect on nucleosome organisation at CTCF binding sites (Fig 4D).

### SNF2H and SNF2L contribute to nucleosome phasing adjacent to the binding sites of many transcription factors

**Fig 5:**
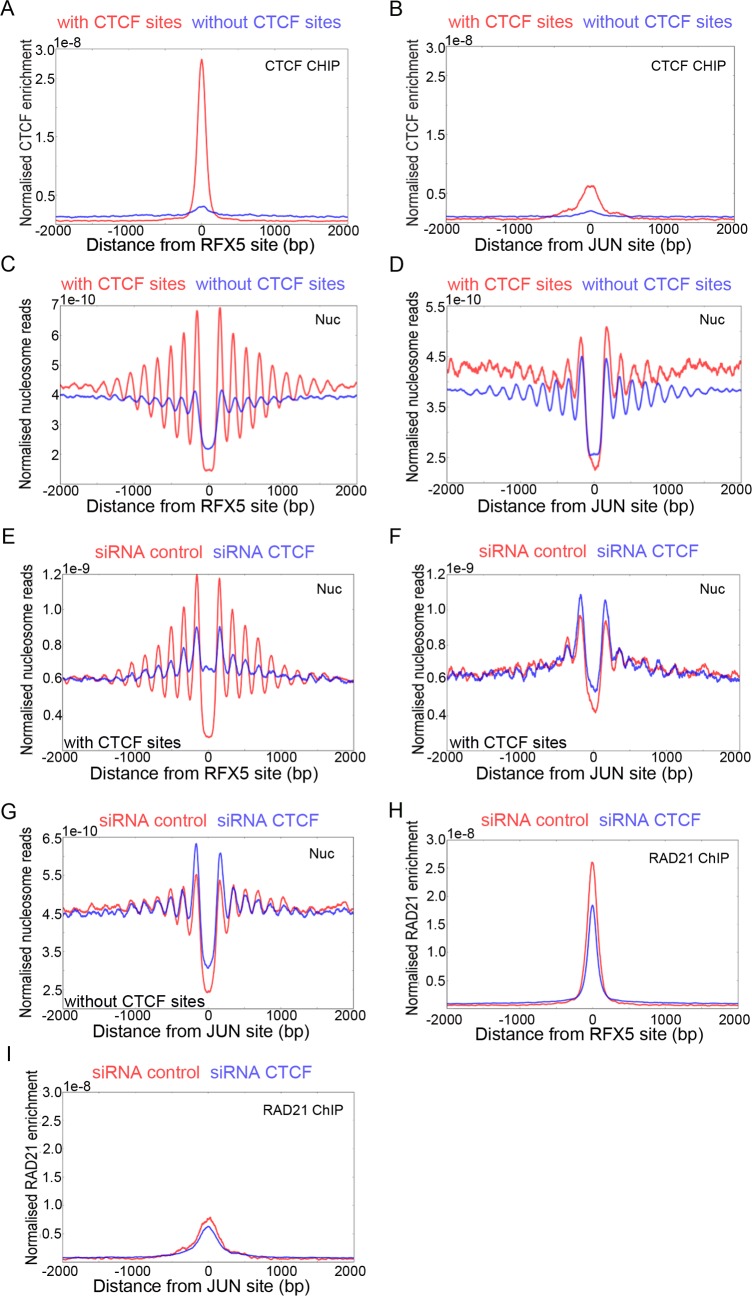
Constructive and destructive interference between nucleosomes organized by CTCF and other transcription factors. (A, B) ChIP seq enrichment for CTCF at sites bound by RFX5 (A) and JUN (B). For each transcription factor, data for subsets of sites that either include (red) or do not include (blue) CTCF bound sites within 500 bp was plotted. The removal of coincident CTCF sites greatly reduces CTCF ChIP at both RFX5 and JUN sites (A, B). Nucleosome density plots for RFX5 sites (C) that include CTCF sites within 500 bp (red) and that do not include CTCF sites (blue) 500 bp show that nucleosomes are better organised at RFX5 binding sites that do have adjacent CTCF sites (C, red). In contrast, at JUN binding sites nucleosomes are better organised at the subset of sites not flanked by CTCF sites (D, blue). Nucleosome density plots for RFX5 (E) and JUN (F, G) after CTCF depletion (blue) and control cells (red). CTCF removal disrupts nucleosome organisation at RFX5 sites (E, blue) and improves it slightly at JUN sites if CTCF sites are adjacent (F, blue). Removing JUN sites where CTCF sites are within 500 bp shows a much more prominent nucleosomal pattern with or without CTCF depletion (G). ChIP seq enrichment for RAD21 at RFX5 (H) and JUN (I) binding sites. RAD21 is enriched at factor binding sites with CTCF sites within 500 bp (red). Depletion of CTCF has limited effect on RAD21 enrichment (blue) at RFX5 and JUN sites, indicateingthat RAD21 enrichment is only partially CTCF dependent at these sites.

Previous studies have collated ChIP data characterising the interaction sites for some 119 different transcription factors [32] and this information can be used to align nucleosome distribution adjacent to these factors [9]. Here we select 50 factors for which there are over 1000 genomic binding sites characterised in HeLa cells. Consistent with previous studies we find that binding sites for some factors are located in regions of nucleosome depletion or enrichment without precise positioning of adjacent nucleosomes, whereas other factors such as JUN and RFX5 are flanked by arrays of positioned nucleosomes (Fig 5C and 5D and S4 Fig).

While performing this analysis we observed that by ChIP, we could detect enrichment for CTCF at the binding sites for many transcription factors (Fig 5A and 5B and S4 Fig). We reasoned that in some cases CTCF binding sites are located adjacent to the binding sites for other factors. To test this we filtered out any factor binding sites that had a CTCF binding sites within 500 bp. When only binding sites that did not have CTCF sites within 500 bp were considered, CTCF enrichment at the remaining sites was greatly reduced (Fig 5A and 5B).

We noticed that the effect of filtering out adjacent CTCF sites had differing effects on the organisation of nucleosomes. RFX5 sites that do include adjacent CTCF sites have well organised arrays of nucleosomes (Fig 5C, red). In contrast the RFX5 sites that are not adjacent to CTCF sites have less well organised adjacent nucleosomes (Fig 5C, blue). For RFX5 this effect is very significant as 38% of RFX5 sites are within 500 bp of a CTCF site. Depletion of CTCF significantly perturbs the organisation of nucleosomes at RFX5 sites with adjacent CTCF sites (Fig 5E). This shows that the correlation between the presence of adjacent CTCF sites is functionally significant for nucleosome organisation. CTCF also contributes to the recruitment of cohesin at RFX5 sites as this is reduced following CTCF depletion (Fig 5H). However, the proportion of Rad21 that remains associated following depletion of CTCF indicates that RFX5 is capable of recruiting some cohesin independently of CTCF.

In contrast to the observations at RFX5 sites, the nucleosomes distal to JUN sites are affected in a more complex way. The two nucleosomes immediately adjacent to JUN sites are better organised when there are nearby CTCF sites whilst the extended array of nucleosomes extending beyond the third nucleosome is less ordered as assessed by the depth and periodicity of the normalised read depth (Fig 5D). CTCF depletion results in a modest improvement to nucleosome organisation at JUN sites with adjacent CTCF (Fig 5F). JUN sites lacking adjacent CTCF sites show less change of the distal nucleosomal array following CTCF depletion (Fig 5G). Depletion of CTCF has only a minor effect on Rad21 ChIP at JUN sites indicating that JUN can organise cohesin independently (Fig 5I).

The effects of adjacent CTCF sites observed at RFX5 sites are also observed at the binding sites for other transcription factors. For example nucleosomes are better organised adjacent to the binding sites of factors such as BRCA1 and GTF2F1 that have adjacent CTCF sites (S4 Fig). Enrichment of cohesin is often affected in a similar way (S5 Fig). This illustrates a pitfall in the use of averaging to study correlations in the distributions of chromatin associated factors at complex regulatory elements which are likely to include binding sites for many different factors. For this reason we consider only factor binding sites that do not have adjacent CTCF sites for the subsequent analysis.

**Fig 6:**
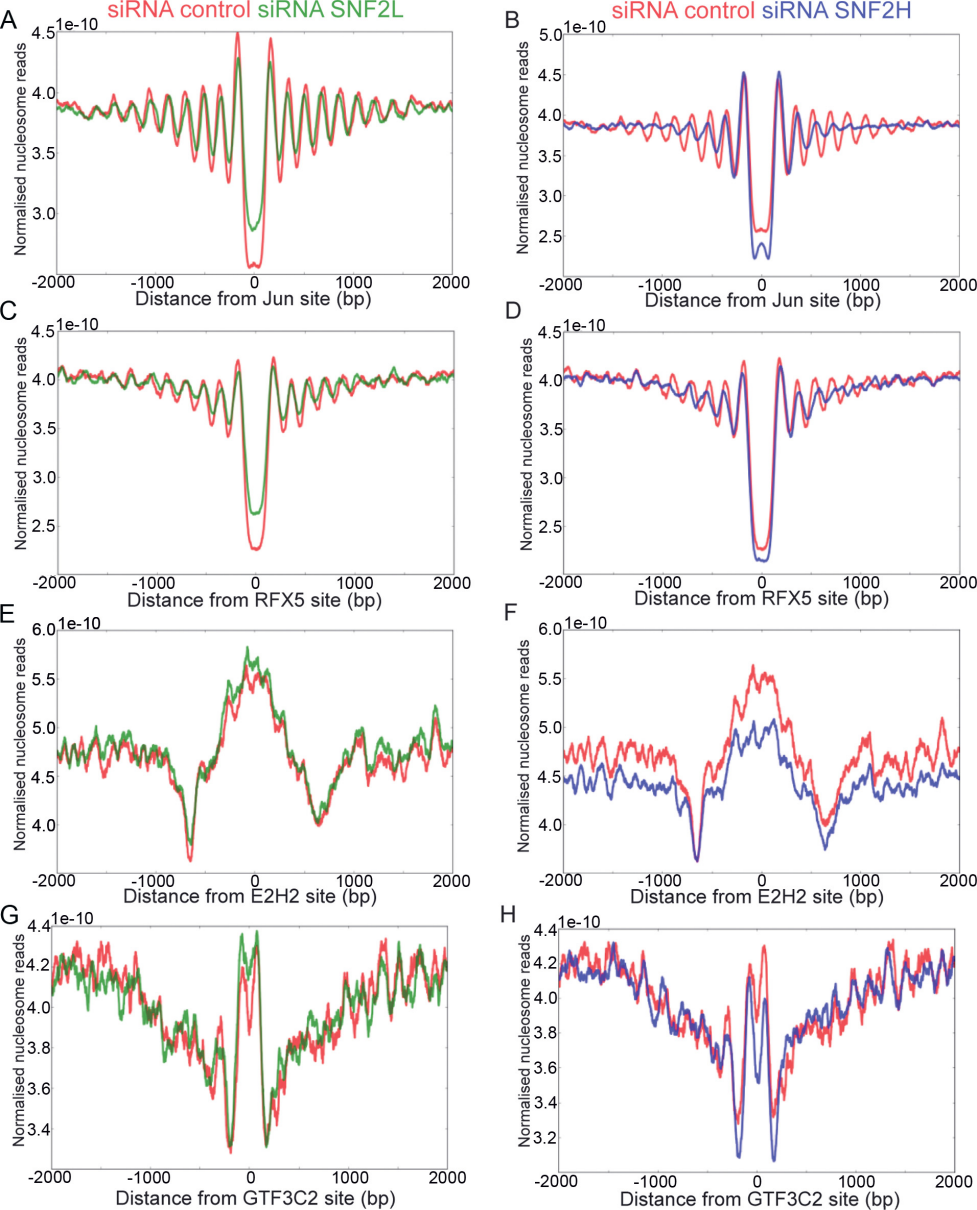
SNF2L and SNF2H contribute to the nucleosome phasing at transcription factor binding sites. (A-D) Nucleosome density plots of sequenced mono nucleosomal DNA after depletion of SNF2L and SNF2H proteins mapped to the JUN (A, B) or RFX5 (C, D) transcription factor binding sites. Depleting SNF2L (A, C green) shows an effect on the factor proximal nucleosomes at majority of transcription factor binding sites while the effect of SNF2H knock down (B, D blue) affects distal nucleosomes at the binding sites for factors such as JUN (see main text). Transcription factors such as E2H2 (E, F) and GTF3C2 (G, H) that have no well organised nucleosomes are less affected by the depletion of SNF2H (F, H blue) or SNF2L (E, G green). In all plots data was only taken from factor binding sites that do not have adjacent CTCF sites.

To investigate the involvement of SNF2H and SNF2L in nucleosome organisation at different factor binding sites, we plotted the organisation of nucleosomes following depletion of each enzyme flanking the binding sites for 50 different transcription factors (S6 Fig). As at promoters significant differences in organisation were observed with different levels of MNase digestion. However differences in chromatin organisation are apparent when compared to control digestions with similar nucleosome fragment lengths. Depletion of SNF2H has effects on nucleosome organisation surrounding binding sites of factors such as JUN (Fig 6B). The effects are most pronounced for nucleosomes distal to the factor binding site. For example the nucleosomes distal to the +3 nucleosome are less well organised at JUN sites following SNF2H depletion (Fig 6B). Similar effects are observed surrounding 24 additional transcription factors (S6 Fig). Depletion of SNF2L was observed to result in a small reduction in occupancy of nucleosomes proximal to a subset of factor binding sites (Fig 6A and 6C and S6 Fig).

For all factors where SNF2H or SNF2L depletion was observed to affect the generation of extended nucleosomal arrays, SNF2H or BPTF were also observed to be present by ChIP (S5 Fig). For example at JUN sites there is enrichment for SNF2H and BPTF by ChIP. However, enrichment for SNF2H and BPTF was also observed at some factor binding sites where nucleosomes are not well organised, for example GTF3C2 (S4 and S5 Figs). Binding of SNF2H was not enriched at all sites, for example as observed at E2H2 and FAM48A sites (S5 Fig) and there is minimal nucleosome organisation at these sites.

### Co-regulation of transcription by SNF2H and CTCF

**Fig 7:**
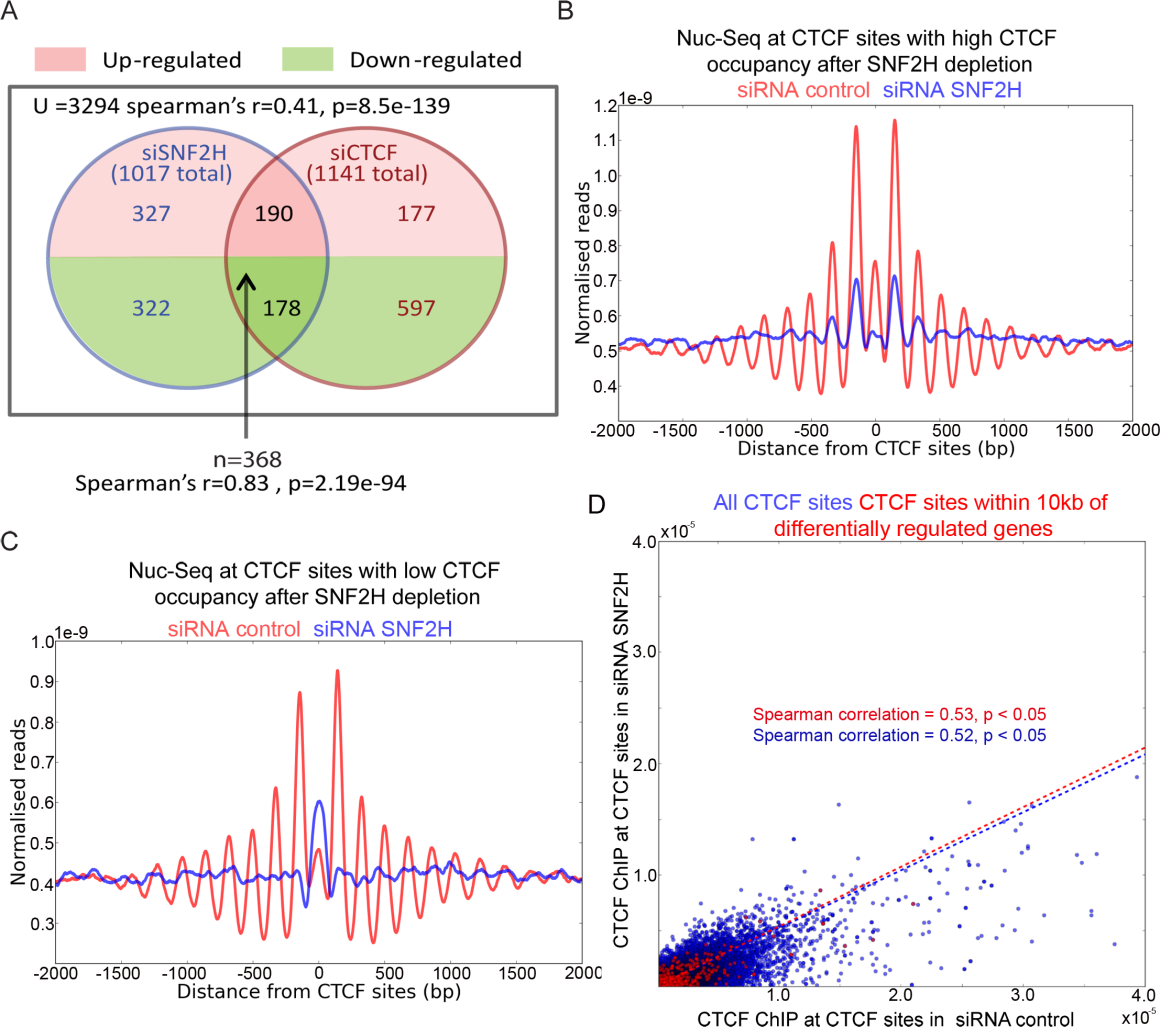
Depletion of SNF2H affects the transcription of many genes regulated by CTCF. (A) RNA seq was performed for cells depleted of CTCF and SNF2H. Expression of 3294 genes could be significantly (p<0.05 and q<0.05) measured across four repeats in both cell types. The total number of affected genes following depletion of CTCF and SNF2H is shown in brackets. There is significant overlap between genes with altered expression (>1.5 fold) following SNF2H and CTCF depletion. Amongst these co-regulated genes there is a high correlation in the extent to which expression is affected in each depletion (Spearman’s correlation 0.83, p = 2.19e-94). This indicates that a significant subset of CTCF regulated genes are co-regulated by SNF2H. (B) Nuc seq of CTCF sites that retain CTCF occupancy (top 10%) following SNF2H depletion (blue) in comparison to control RNA depletion (red). Despite the retention of CTCF nucleosome organisation is reduced at these locations in the absence of SNF2H. (C) Nuc seq of genes where CTCF occupancy is reduced following SNF2H depletion (blue) in comparison to scrambled RNA depletion (red). At these sites a more dramatic loss of nucleosome positioning is observed following loss of both CTCF and SNF2H. (D) CTCF ChIP following depletion of SNF2H and control scrambled RNA is shown for all CTCF sites (blue) or CTCF sites within 10kb of 368 genes identified as being co-regulated by CTCF and SNF2H (as described in A). Least squares fits and Spearman correlation for each data series are also shown. The changes in CTCF occupancy at sites within the vicinity of co-regulated genes is very similar to that observed for all other sites. This indicates that changes in CTCF occupancy following SNF2H depletion do not provide a simple explanation for co-regulation by SNF2H and CTCF.

To investigate the functional significance of SNF2H dependent phasing of nucleosome arrays we compared the effects of depleting CTCF and SNF2H. Approximately 1000 genes were significantly affected by the transient depletion of either protein. Many of the up and down regulated genes are affected similarly by depletion of CTCF or SNF2H (Fig 7A). This overlap is highly statistically significant with P values lower than 10^−50^. The most probable explanation for this is that SNF2H is required for the function of a significant subset of CTCF sites. As SNF2H affects both CTCF occupancy and nucleosome positioning it is difficult to distinguish which is dominant. However, it is possible to identify cohorts of genes where CTCF occupancy was either unchanged following SNF2H depletion or reduced. At the genes where CTCF is retained nucleosome positioning is reduced following SNF2H depletion (Fig 7B). In contrast, where CTCF occupancy is lost, nucleosome organisation is completely lost (Fig 7C). To investigate whether changes of CTCF occupancy were correlated with genes that showed changes in expression following depletion of SNF2H, occupancy of CTCF was assessed at all sites within 10kb of genes that changed expression. The changes in CTCF occupancy at genes that changed expression were indistinguishable from the changes observed at all genes (Fig 7D). This suggests that the overlap between genes affected by CTCF and SNF2H depletion cannot accounted for by a simple change in CTCF occupancy.

## Discussion

Previously it has been observed that nucleosomes are organised as phased arrays adjacent to the binding sites of a subset of metazoan transcription factors [9] and at promoters [5, 33]. However, the factors responsible for the organisation of these arrays have not been established. Here we have systematically investigated the contributions of candidate remodelling ATPases in chromatin organisation. At promoters we did not identify a role for any individual enzymes in organising nuclesomes. Consistent with this changes to nucleosome organisation surrounding the binding sites for general transcription factors such as TBP and TAF1 were only modestly affected by depletion of SNF2H or SNF2L (S6 Fig). Our attempts to pursue this further through depleting combinations of remodellers simultaneously did not provide evidence for this. It is possible that the siRNA strategy was less effective at depleting multiple enzymes or that additional as yet unidentified factors contribute to nucleosome organisation at promoters.

During the course of the study we became aware that the distribution of reads surrounding promoters and factor binding sites is sensitive to the extent of MNase digestion. A similar effect has been observed at promoters in yeast [34]. It could arise from differences in the accessibility of nucleosomes in different locations and or differences in the stability of nucleosomes to higher levels of digestion. The end result is that nucleosomes flanking many factor binding sites are enriched at low in comparison to high levels of digestion. Following sequencing a good way to assess the extent to digestion is to measure the mean nucleosomal read length as this decreases with increasing digestion (S1 Table). We sought to ensure that the difference between these mean read lengths was within 5 bp between control and experimental depletions.

### SNF2H plays a major role in the establishment of extended nucleosomal arrays at CTCF sites

At CTCF sites we observed that depletion of SNF2H resulted in a substantial reduction to nucleosomal pattern flanking these sites. This establishes that SNF2H plays the major role in the establishment of the remarkably well organised arrays of nucleosomes observed flanking CTCF sites. Several additional remodelling ATPases including CHD4, CHD8 and SNF2L have also been reported to be recruited to CTCF sites [25, 26, 28]. These enzymes are unlikely to have major roles in the establishment of extended nucleosomal arrays adjacent to CTCF sites as this is so strongly dependent on SNF2H. Depletion of SNF2L or BPTF had minor effects on the nucleosomes proximal to CTCF sites. This localised effect in the SNF2L depletion is consistent with a local alteration to digestion detected using microarrays [28].

The SNF2H ATPase is present within multiple distinct complexes. The effects of depleting distinguishing subunits of these complexes were inconclusive suggesting that there may be some redundancy between different complexes. However, depletion of the ACF1 subunit of the SNF2H containing human ACF complex and the WSTF subunit of the human WICH complex resulted in subtle reductions to nucleosome organisation especially at locations distal to CTCF (S3 Fig). Both the ACF and WSTF complexes have the biochemical capability to organise chromatin [35, 36].

SNF2L has been purified as a component of a distinct remodelling complexes, NURF [31] and CERF [37]. Expression of SNF2L was originally thought to be restricted to brain and gonadal tissue [38] however, more recent studies indicated that it is ubiquitously expressed [39] and has functions in Wnt signalling and at CTCF sites [28, 39, 40]. The CERF complex is found in neural tissues [37]. The biochemical activities of NURF are distinct from those of ACF in that NURF was originally purified based upon its ability to disrupt nucleosomal arrays [30]. The role that human NURF plays in nucleosome positioning adjacent to human transcription factors is consistent with the original assays for DNaseI hypersensitivity. Although NURF repositions nucleosomes, it also interacts with transcription factors [41] and this can result in directional repositioning of nucleosomes adjacent to factor binding sites [42]. Thus, the NURF complex has the biochemical properties to direct the positioning of nucleosomes immediately adjacent to factors such as CTCF.

### SNF2L and SNF2H contribute to nucleosome phasing adjacent to a disparate range of transcription factors

We also investigated the effects of chromatin remodelling enzymes on nucleosome organisation at the binding sites for 49 additional transcription factors and at promoters. 29 of these organise extended arrays of nucleosomes and SNF2H contributes to nucleosome organisation at most of these (24/29)(S6 Fig). Typically the nucleosomes immediately flanking the factor binding site are best organised. The distance between these +1 and -1 nucleosomes flanking factor binding sites ranges from 258 bp (REST) to 364 bp (RCOR1). This is substantially larger than would be anticipated based on steric occlusion at the factor binding site and the linker observed between adjacent nucleosomes. It may be that many of these factors are bound by additional cofactors. For example, CTCF is known to associate with TAF3 [43]. Following depletion of SNF2H the distance between the +1 nucleosomes flanking factor binding sites increasing by on average 25bp, in addition the average separation between flanking nucleosomes increases from 176 bp to 183 bp. This is consistent with a role for SNF2H in driving nucleosomes together and towards the factor binding site. The effects following SNF2L depletion are relatively minor, but also distinct in that the distance between adjacent +1 nucleosomes reduces by 10bp and the separation between adjacent nucleosomes is reduced from 176 to 173 bp. This suggests that SNF2L complexes may act to move nucleosomes away from bound factors. The finding that different remodelling enzymes act to alter nucleosome positioning with different directionality is reminiscent of the way remodelling enzymes act with different directionalities at yeast promoters [44] and suggests that a similar interplay operates at the binding sites for a range of transcriptional regulators. With the possible exception of SNF2H at CTCF sites, the effects of depleting enzymes result in alterations to the distributions of nucleosomes rather than complete loss. This suggests that as yet unidentified factors are likely to function in a partially redundant fashion with SNF2H and SNF2L.

*In vitro*, it has been observed that bound transcription factors act as a barrier restricting the positioning of nucleosomes remodelling enzymes [45]. The observations made here provide evidence that the biophysical interplay between bound factors and nucleosome repositioning characterised *in vitro* is likely to contribute to nucleosome organisation at functional regulatory elements. Nucleosomes positioned adjacent to such barriers could act as a reference point from which progressively distal nucleosomes are organised [46], potentially providing a means of organising chromatin adjacent to any bound factor. This raises the question why are nucleosomes much better organised adjacent to some bound factors than others?

It is possible that targeted recruitment of remodelling enzymes is required in addition to the presence of a barrier. For example both SNF2L and SNF2H interact with CTCF [27, 28]. However, we also observe dependency upon SNF2L and SNF2H at the binding sites for an additional 24 transcription factors. It is difficult to imagine that SNF2H and SNF2L containing complexes possess the capability of recognising such a structurally diverse range of factors. For this reason we consider it likely that in addition to direct association with transcription factors other interactions contribute to the recruitment of these enzymes.

A prime candidate would be modification to histones such as H3 K4 trimethylation which is enriched at the binding sites for many transcription factors [9]. The SNF2L containing NURF complex has specificity for histone H3 methylated at lysine 4 [47] and so this modification is likely to contribute to recruitment. In budding yeast, ISWI chromatin remodelling enzymes have been shown to be recruited by a looping mechanism [48]. As CTCF sites are also sites of gene looping, this mode potentially provides an additional means via which human ISWI containing enzymes could be recruited.

### Adjacent factor binding sites cooperate to organise chromatin

Eukaryotic regulatory elements seldom consist of the binding sites for a single sequence specific regulatory factor in isolation. Instead binding sites for disparate factors are often found within close proximity. This complicates the interpretation of which chromatin features are directly recruited by a transcription factor and which are influenced by neighbouring factors. Our own data show that both nucleosome organisation and cohesin enrichment at many factor binding sites is influenced by the presence of adjacent CTCF sites (S4 and S5 Figs). CTCF is an unusual transcription factor in that its interaction with DNA is very stable [49] and it plays a major role in determining the distribution of cohesin during interphase [15–18]. This means that interference from adjacent CTCF sites may have especially strong effects. Nonetheless, in principle, similar forms of interference could occur between any two transcription factors and affect the distribution of any chromatin feature such as a histone modification or a cofactor. The scope for misinterpretation as a result of this type of interference is especially high when averaged enrichment is considered for many sites. The use of averaging in metazoan genomic datasets is often essential as the read depth with which data has been collected is in many cases not sufficient for analysis at single genes. This is especially relevant for high resolution studies of nucleosome positioning as this requires a high read depth at each base pair. Obtaining data with the required depth is in most cases impractical and averaging at many related sites provides a way round this. To our knowledge, there is one dataset, with a depth of 3.6 billion reads, that potentially does have the depth required to call nucleosome locations at single loci in human cell lines [50]. However, we could not use this to study alignment to CTCF or other transcription factors as ChIP has not been performed to determine the occupancy of these factors in the cell lines used.

The enhanced nucleosome phasing adjacent to sites such as RFX5 and ZNF143 that have adjacent CTCF sites is best explained if these different factors constructively interfere with each other to generate stronger nucleosome phasing (Fig 5 and S4 Fig). This would require that the binding sites are in phase with the nucleosomal repeat. A large proportion of the CTCF sites are immediately adjacent to RFX5 and ZNF143, so this is feasible. At JUN sites nucleosome organisation increases following removal of CTCF sites in silico or following depletion of CTCF (Fig 5 and S4 Fig). This suggests that in this case CTCF destructively interferes with the phasing of nucleosomes established by JUN. Constructive and destructive interference in the phasing of nucleosomes by different factors has also been observed on adjacent coding genes in yeast [51]. These findings indicate the potential for complexity in the way that chromatin is arranged over regulatory elements that contain binding sites for many different factors.

### Nucleosome phasing as a means to promote factor occupancy

We observed that depletion of SNF2H results in a reduction in the occupancy of CTCF at many sites (Fig 3). Consistent with this depletion of SNF2H has previously been observed to result in decreased binding of CTCF at the H19/Igf2 locus [27]. This suggests that the action of SNF2H promotes CTCF binding. There is a literature supporting a role for ATP-dependent remodelling enzymes in facilitating the binding of transcription factors to chromatin [52] however, this has typically involved enzymes such as SWI/SNF that disrupt chromatin organisation rather than ISWI containing enzymes such as ACF that space nucleosomes evenly. How then could a nucleosome spacing enzyme act to promote factor occupancy? Currently favoured mechanisms for nucleosome spacing involve the enzyme sensing DNA adjacent to nucleosomes such that repositioning occurs towards the side of a nucleosome with a long accessible linker [23]. This results in the repositioning of nucleosomes with a mean location equidistant between neighbouring nucleosomes. Strongly bound transcription factors such as CTCF also potentially reduce access to linker DNA. In this situation a spacing enzyme would be anticipated to move a nucleosome away from the factor bound linker. Indeed the positioning of nucleosomes by ISWI-related complexes has been observed to be affected by transcription factor binding *in vitro* [53, 54]. The repositioning of nucleosomes away from factor bound sites effectively partitions DNA sequences occupied by transcription factors and nucleosomes. As a result of reduced competition with nucleosomes the factor bound state would be favoured. This contrasts with the action of complexes such as SWI/SNF which move nucleosomes across factor binding sites resulting in dissociation [54].

Reducing competition with nucleosomes may be especially important at CTCF sites as the binding consensus sequence has high GC content and high inherent affinity for nucleosomes [9]. Therefore, unbound CTCF sites are likely to be occupied by nucleosomes. Supporting this increased nucleosome occupancy is observed at CTCF sites that are only occupied in specific cell lines [55] and in our own data following depletion of CTCF or SNF2H (Figs 2A, 2B and 7C). On the other hand when bound by CTCF the action of SNF2H acts to reduce competition with nucleosomes and further stabilise the bound state. The positive feedback favouring both bound and non-bound states may help to explain how the subset of CTCF consensus sequences that are actually bound varies between different cell lines [55] and during differentiation [56].

Following depletion of SNF2H a quite striking increase in the occupancy of nucleosomes well positioned over CTCF sites is observed at locations where CTCF occupancy is reduced (Fig 7C). These well positioned nucleosomes do not by themselves result in the establishment of well-ordered arrays of flanking nucleosomes. This suggests that the level of non-targeted nucleosome spacing activity in human cells is insufficient on its own to establish ordered arrays of nucleosomes. At the sites where well-ordered arrays are observed, there is likely to be a requirement for both a barrier from which the array is established and targeted recruitment of enzymes such as SNF2H and SNF2L to propagate and maintain spaced chromatin.

### The role of SNF2H in cohesin recruitment

As CTCF acts to recruit cohesin and SNF2H promotes CTCF occupancy, SNF2H would be expected to influence cohesin occupancy at CTCF sites. This is indeed the case as we observe a reduction in cohesin by ChIP at CTCF sites following SNF2H depletion (Fig 4). A previous study also observed that loading of cohesin was reduced following inactivation of SNF2H [57]. To investigate whether SNF2H contributes to cohesin loading independently of its effect on CTCF binding, the enrichment of Rad21 was plotted at CTCF sites that remain occupied and adjacent to the binding sites for other transcription factors. Enrichment for Rad21 is not affected at these locations following depletion of SNF2H (S7 Fig). This suggests that SNF2H is not a general loading factor for cohesin, but affects its loading at a subset of CTCF sites. We do not believe that a remodelling complex containing cohesin contributes to nucleosome organisation at CTCF sites as depletion of Rad21 has no effect on nucleosome organisation (Fig 4D).

### SNF2H function at CTCF sites

Following SNF2H depletion we observe that nucleosomes become disorganised and CTCF occupancy is reduced. As many CTCF dependent genes show changes in expression following SNF2H depletion, in principle either or both of these effects could contribute to SNF2H function. However, the lack of any difference in CTCF occupancy at the CTCF target genes affected by SNF2H depletion (Fig 7D) suggests that changes in CTCF occupancy do provide a simple means of explaining the effects on transcription. This raises the possibility that nucleosome positioning is functionally significant, but further investigation will be required to establish this rigorously.

The internucleosome spacing adjacent to CTCF sites is 176 bp, 19 bp shorter than the major internucleosome spacing of 198 bp detected in mammalian cells [2]. In addition, the nucleosomes adjacent to CTCF binding sites are unusually well translationally positioned. The presence of similarly well organised nucleosomes over yeast coding genes is correlated with low histone turnover, histone modification and reduced non coding transcription [58–60]. As non-coding transcription also contributes to enhancer function [61] it is possible that the organised nucleosomes adjacent to CTCF sites also affect enhancer function via RNA mediated pathways.

## Materials and Methods

### Cell culture

HeLa cells originally obtained from the ATCC Global Bioresource Center were cultured in DMEM (Invitrogen) supplemented with 0.2 mM l-glutamine and 10% FBS.

### RNA interference

The siRNA oligonucleotides were purchased from Eurofins MWG used at a final concentration of 7.8 nM. The siRNA transfections were performed using INTERFERin (Polyplus Transfections). All siRNA sequences are listed in Table 1. Cells were transfected three times according to the INTERFERin protocol with 72hours of growing in between transfections.

**Table 1:** siRNA Oligos Used for RNA Interference.

**Protein**	**Oligo**	**Sequence 5’ to 3’**
hSNF2H	siSNF2H	AGAUGAGAAGCAGAACUUATT
hSNF2H	siSNF2HB	GGGCAAAUAGAUUCGAGUA
CTCF	siCTCF	GGAGCCUGCCGUAGAAAUUTT
RAD21	siRAD21	GGUGAAAAUGGCAUUACGGTT
CHD1	siCHD1	UCAUAAACCAACACAGUAATT
CHD2	siCHD2	AAAAGCAAGAUUCUUCUGAUGAGGAUG
CHD4	siCHD4	CCCAGAAGAGGAUUUGUCATT
hSNF2L	siSNF2L	GGAAAUGGACCCAGAAUAUTT
ACF1	siACF1	GCAACACUGUGAACCACAATT
WSTF	siWSTF	GGAAGGAGAGAGAGUAUUATT
TIP5	siTIP5	UCACUGAGCUAUUGAACAATT
MTA2	siMTA2	CCACAGACCGGUAUAUUCATT
BPTF	siBPTF	CUGAAGACCUGACCAAUAATT
Scramble	siScr	AACAGUCGCGUUUGCGACUGGTT

### Whole cell extract preparations

To check for depletion of proteins after siRNA transfections, whole cell extracts of HeLa cells were prepared by lysing cells in WCE-buffer (20mM Hepes pH7.6, 400mM NaCl, 1 mM EDTA, 25% glycerol, 0.1% NP-40, protease inhibitors) followed by homogenization using a syringe. SNF2L depletion was checked by directly lysing counted cells using urea sample buffer as described by Eckey M. et al, [39].

### Antibodies

Primary antibodies for Western blots used were rabbit anti-human SNF2H (Bethyl Laboratories, A301-081A), mouse anti-CTCF (Abcam, ab37477), rabbit anti-RAD21 (Abcam, ab992), rabbit anti-CHD1 (Bethyl Laboratories, A301-218A), rabbit anti-CHD2 (Active Motif, 39364), rabbit anti-CHD4 (Bethyl Laboratories, A301-081A), rabbit anti-ACF1 (Bethyl Laboratories, A301-318A), rabbit anti-WSTF (Cell Signaling, 2152), rabbit anti–TIP5 (Invitrogen, 491037) and mouse anti-beta actin (Sigma, A2228). Primary antibodies for ChIP used were rabbit anti-human SNF2H (Abcam, ab72499), mouse anti-CTCF (Millipore, 17–10044), rabbit anti-RAD21 (Abcam, ab992), rabbit anti-BPTF (Millipore, Abe24), and rat anti-SNF2L (2C4, [39] which has been kindly provided by P. Becker). Secondary antibodies for Western blots used were Alexa Fluor 680 goat anti-mouse (Invitrogen) and Alexa Fluor 790 goat anti-rabbit (Invitrogen) for immunofluorescence staining and analysis using the LI-COR Odyssey CLx.

### Real time qPCR

For verification of the RNAi depletion of TIP5, and SNF2L RNA was isolated using the QIAGEN RNeasy kit according to the manufacturer. 2μg RNA was reverse transcribed into cDNA (QIAGEN, QuantiTect kit). PCR was carried out in a total volume of 15 μl by using 2 μl of cDNA with the Quanta PerfeCTa SYBR Green FastMix and TIP5 transcript primers ((1) for TTCTCCTATGTTGGGATCTAGCA/ rev CAGTGCCATTCTCTGCCACA and (2) for GGCCTACGACTGTCTCTGGAA/ rev TTGGGGATGAAGGTTGCCG) or SNF2L transcript primers ((1) for AAGCGCCTAAATATGAAAAGGA/ rev GCGGTAGTCTCCAGCAGAAAT and (2) for GCTGGAGACTACCGCCCATAG/ rev CAACCAATTCAGTAATCGAATAT) according to Quanta standard protocol using an AB 7500 Real Time PCR Cycler. Beta-actin transcript was used for normalization.

### MNase digests

_~_8 x10^5^ siRNA-transfected cells were crosslinked with 1% formaldehyde for 10 min and quenched for 5 min with 125 mM glycine at room temperature. After washing cells with cold PBS, cells were lysed using cold NP40-lysis buffer (10 mM Tris pH 7.5, 10 mM NaCl, 3 mM MgCl_2_, 0.5% NP40, 0.15 mM spermine, 0.5 mM spermidine) for 5 min on ice. Cells were pelleted and washed with MNase digestion buffer (10 mM Tris pH 7.5, 15 mM NaCl, 60 mM KCl, 1 mM CaCl_2_, 0.15 mM spermine, 0.5 mM spermidine) and resuspended in 50 μl MNase digestion buffer. For the digest, 3units MNase S7 (Roche) were added and incubated for 2 min (low digest) or 4 min (high digest) at 37°C. The digest was stopped adding 1/10 vol 10% SDS and 1/10 vol 250 mM EDTA. NaCl was added at a final concentration of 0.2M to reverse the crosslinking at 65°C overnight. The samples were treated with 40 μg proteinase K for 30 min at 45°C and 10 μg RNase A for 30 min at 37°C, followed by phenol-chloroform extraction and purification using a PCR purification kit (QIAGEN). The samples were eluted from the columns with 50 μl 10 mM Tris pH 7.5 and run on a 1.2% agarose gel in 1 x TAE. The gels were stained using SYBR Safe DNA gel stain (Invitrogen) and mono nucleosomes were cut out. Bands were gel extracted with the QIAGEN gel extraction kit. The resulting DNA fragments were used for Illumina library preparations.

### Chromatin immunoprecipitation

_~_3.2 x10^7^ siRNA-transfected cells were used per ChIP. Cells were cross-linked with 1% formaldehyde for 10 min and quenched for 5 min with 125 mM glycine at room temperature. After washing cells with twice with ice-cold PBS, cell pellets were flash frozen in liquid nitrogen and stored at -80°C. Frozen cell pellets were lysed in 1.8 ml lysis buffer containing 1% SDS, 10 mM EDTA, 50 mM Tris pH 8.1 and protease inhibitors. To shear chromatin to fragments of about 200–500 bp size, samples were sonicated in 300 μl volumes for 15 cycles (7.5 min total sonication time) at high setting using a Bioruptor (Diagenode). Sonicated lysates were then cleared by centrifugation for 10 min at high speed, diluted 1/10 with dilution buffer (1% Triton X-100, 2 mM EDTA, 150 mM NaCl, 20 mM Tris at pH 8.1, 0.1% Brij-35) and incubated with 12 μg of the respective antibody overnight at 4°C. For each ChIP, 200 μl of Protein G Dynabeads (Life Technologies) were pre-incubated with 0.5% (w/v) BSA in PBS overnight. To capture antibody-bound protein-DNA complexes, lysates were incubated with the prepared beads for 3 hrs and subsequently washed twice with 6ml of each wash buffer I (0.1% SDS, 1% Triton X-100, 2 mM EDTA pH 8.0, 20 mM Tris pH 8.1, 150 mM NaCl), wash buffer II (0.1% SDS, 1% Triton X-100, 2 mM EDTA pH 8.0, 20 mM Tris pH 8.1, 500 mM NaCl) wash buffer III (0.25 mM LiCl, 1% NP-40, 1% sodium deoxycholate, 1 mM EDTA pH 8.0, 10 mM Tris pH 8.1) and TE buffer (10 mM Tris pH 8.1, 1 mM EDTA pH 8.0) in the cold. To elute, reverse-crosslink and purify ChIP DNA the IPure kit from Diagnode was used according to the manufacturer. The resulting DNA was used for Illumina library preparations.

### Illumina library preparation

Libaries from ChIP DNA or mono nucleosomal DNA resulting from MNase digests were prepared using the protocol published (Bowman SK et al, BMC Genomics 2013) with modifications. All enzymes, buffers and nucleotides were purchased from Fermentas unless stated differently. In short, DNA was end repaired in 50 μl reactions containing 1x T4 ligase buffer, 0.4 mM dNTPs, 7.5 U T4 DNA polymerase, 5 U Klenow polymerase, 15 U T4 polynucleotide kinase for 30 minutes at room. To purify DNA, 1.8 volumes Agencourt AMPure XP beads were used according to the manufacturer. A-tailing reactions (50 μl) contained cleaned up DNA, 1x Klenow buffer, 2 mM dATP, 15U Klenow 3’-5’ exo—and were incubated for 30 minutes at 37°C. DNA purification was performed using 1.8 volumes Agencourt AMPure XP beads. Adapter ligation reactions in 50 μl volumes contained DNA from A-tailing, 1x T4 ligase buffer, 0.04 μM annealed universal adapter for ChIP samples and 2 μM adapter for mono nucleosomal DNA, 5 Weiss U T4 ligase and were incubated overnight at 16°C. This time DNA was purified using 1.1 volumes of Agencourt AMPure XP beads to avoid co-purifying excess adapter. DNA was eluted using 20 μl H_2_O of which half was used to amplify DNA in the next step.

For library amplification the PCR reactions contained 5 μl adapter ligated DNA, 1x Phusion HF buffer (Thermo Scientific), 0.3 μM Illumina universal primer, 0.3 μM Illumina barcoded primer, 0.4 mM dNTP, 200mM Trehalose and 3 U Phusion Hot Start II High Fidelity DNA Polymerase (Thermo Scientific). Thermocycling was performed by denaturing for 3 minutes at 98°C; followed by 20 cycles for ChIP DNA and 10 cycles for mono nucleosomal DNA of: 15 seconds at 98°C, 25 seconds at 60°C, and 1 minute at 68°C, and a final extension of 5 minutes at 68°C.

PCR products were resolved on a 1.2% agarose gel in 1x TAE. _~_250 bp to 700 bp of ChIP DNA and _~_300 bp of mono nucleosomal DNA was extracted using QIAGEN MinElute Kit and sent for sequencing.

### Nuc seq and ChIP seq data analysis

Paired end libraries of MNase digested chromatin ChIP DNA were sequenced using illumine HiSeq technology. Fastq files containing raw reads were aligned to human reference genome (ftp://ftp.ccb.jhu.edu/pub/data/bowtie2_indexes/hg19.zip) hg19 by Bowtie2 with option of maximum fragment length of 1500 for chip data and 500 for nucleosome fragments. Fragment length distributions for each sample used are shown in S1 Table. The midpoints of uniquely mapped nucleosomal or ChIP reads were used for further analysis.

Transcription factor tracks [62] in HeLa cells were downloaded using the UCSC table browser [63] of the encode database (http://genome.ucsc.edu/ENCODE/dataMatrix/encodeChipMatrixHuman.html) as narrow Peak file formats. A 2kb region flanking the TFB site was selected for nucleosome or ChIP enrichment analysis. The nucleosome dyads/chip fragment reads coverage was calculated for each base in the 2kb region. This enrichment value at each base was then divided by number of TFB sites and total number of reads in the experiment to obtain normalised reads. The plotted data was normalised to have same mean read counts in the plotted window. The data was smoothed using a 50 bp sliding window for graphical representation. Plots were generated with python’s plotting modules matplotlib and pylab.

All of the data shown in the manuscript was established as being reproducible between repeats of genome wide experiments. In most cases the data plotted is the average of appropriately digested biological repeats. A full description of the data included in each figure is provided in (S1 Table). In some figures the same control enrichments are re-plotted in different panels.

Sequence data is accessible at the European nucleotide archive (ENA) http://www.ebi.ac.uk/ena/data/view/PRJEB8713 under accession number PRJEB8713.

### RNA seq analysis

RNA seq analysis pipeline described in [64] was followed for mapping and measuring differential expression of genes. In brief, the paired end reads of each biological replicate was mapped to hg19 human reference genome independently with TopHat [tophat2 -p 8 -r 200 -g 2 -o output folder hg19 reads_R1_001.fastq reads_R2_001.fastq]. The assembled reads were the provided as input in Cufflinks which generates assembled transcripts for each replicate [cufflinks -p 8 -g hg19_genes.gtf -o output folder mapped reads.bam]. Mapped reads were the used as input in Cuffdiff to obtain differential expression results in tabular format [cuffdiff -p 8 -o output cuffdiff hg19_genes.gtf siScr_1.bam, siScr_2.bam siSNF2H_1.bam, siSNF2H_2 bam]. Transcripts having >1.5 fold changes in their expression were selected as differentially regulated and have uncorrected p-value of the test statistic <0.05 and FDR-adjusted p-value of the test statistic <0.05 were used for further analysis.

## Supporting Information

S1 Fig Effects of depleting CHD1, CHD2 and CHD4 on promoter nucleosome organisation. Nucleosomal reads aligned to the promoters of ubiquitously expressed promoters following depletion of CHD1 (A, blue), CHD2 (B, blue) and CHD4 (C, blue). The red graph depicts the control knock down using a scramble oligo (A, B, C, same in all panels). Depletion of these enzymes has minor effects on the organisation of nucleosomes at promoters. (D) Western blot showing siRNA knock down of CHD1, CHD2 and CHD4 compared to control knock down using a scramble oligo. Level of depletion was determined following normalization to a beta-actin loading control. (E) Isolation of mono nucleosomal DNA fragments following siRNA depletion. Agarose gel showing the DNA fragment length distribution obtained after higher levels of MNase digestion following depletion of the enzymes indicated. The mono nucleosome length species was gel purified and processed for sequencing.
(PDF)

S2 Fig Effects of depleting CHD1, CHD2 and CHD4 on nucleosome organisation adjacent to CTCF binding sites. Nucleosome density plots of sequenced mono nucleosomal DNA fragments after depletion of CHD1 (A), CHD2 (B), and CHD4 (C) proteins aligned to CTCF binding sites. Knock down of the indicated proteins results in relatively subtle changes to the nucleosomal profile.
(PDF)

S3 Fig Depletion of subunits of SNF2H and SNF2L containing complexes has minor effects on nucleosome organisation adjacent to CTCF binding sites. (A)Western blot showing siRNA knock down of ACF1, RSF1, WSTF and BPTF proteins compared to control knock down using scramble oligo. Level of depletion was determined using infrared fluorescence normalised to a beta-actin loading control. Antibodies used as indicated. Due to the lack of a functional antibody, TIP5 depletion of 68% was measured using real time qPCR using two different amplicons. (B-F) Nucleosome density plots of sequenced mono nucleosomal DNA after depletion of SNF2H complex subunits ACF1 (B), RSF1 (C), WSTF (D) and TIP5 (E) proteins and NURF complex subunit BPTF (F) mapped to CTCF binding sites. Knock down of the SNF2H complex subunits result only in minor changes to the distribution of nucleosomal reads while the knock down of BPTF shows a stronger effect on nucleosome occupancy at CTCF binding sites.
(PDF)

S4 Fig CTCF sites interfere with the nucleosome organization at transcription factor binding sites. The removal of coincident CTCF sites greatly reduces CTCF occupancy determined by ChIP at the binding sites for a disparate range of transcription factors. CTCF ChIP seq was plotted at 50 transcription factor binding sites including all sites (red) or sites with CTCF sites within 500bp removed (blue). Nuc seq at 50 transcription factor binding sites was plotted with (red) and without (blue) CTCF sites within 500bp. For many different transcription factors adjacent CTCF binding sites contribute to the nucleosome organisation observed when averaging all sites.
(PDF)

S5 Fig Enrichment of chromatin remodelling enzymes and cohesin at the binding sites for different transcription factors. ChIP seq data for SNF2H and BPTF (first and third panel) at 50 transcription factor binding sites for which at least 1000 bound sites in HeLa cells were identified previously. Second and fourth panel show RAD21 ChIP seq data at factor binding sites plotted with (red) and without (blue) CTCF sites within 500 bp.
(PDF)

S6 Fig Nucleosome organisation adjacent to different transcription factors. Nucleosome seq indicating the positioning of nucleosomes adjacent to 50 transcription factor binding sites after depletion of SNF2L and SNF2H after low (169 bp average nucleosome fragment length) or high MNase digestion (147 bp average nucleosome fragment length). Plots for all 50 factors for which ChIP data identifying at least 1000 bound sites in HeLa was available. The red plots are control knock downs using a scramble oligo while the green plots show SNF2L depletions and blue plots show SNF2H depletions. In all cases data was only taken from factor binding sites that do not have adjacent CTCF sites.
(PDF)

S7 Fig SNF2H depletion does not change RAD21 occupancy at most factor binding sites. ChIP seq data for RAD21 ChIP after SNF2H depletion for 49 factor binding sites. RAD21 enrichment is shown after SNF2H depletion (blue) and in control cells (red). The removal of SNF2H has no effect on RAD21 enrichment at these factor binding sites which contrasts with the effect observed at CTCF sites shown in Fig 4B.
(PDF)

S1 Table Summary of sequence datasets generated for this study. The most abundant read length, read depth and anticipated coverage are indicated for each sequence dataset generated for this study. Also included is a description of which datasets are plotted in each figure.
(PDF)
