# Correction: The Chromatin Remodelling Enzymes SNF2H and SNF2L Position Nucleosomes adjacent to CTCF and Other Transcription Factors

**DOI:** 10.1371/journal.pgen.1006086

**Published:** 2016-05-24

**Authors:** The PLOS Genetics Staff

There is an error in the designation of equal author contributions. The first two authors, Nicola Wiechen and Vijender Singh, should be noted as contributing equally to this work. The publisher apologizes for the error.
