# Supplementary figures and images for: The Chromatin Remodelling Enzymes SNF2H and SNF2L Position Nucleosomes adjacent to CTCF and Other Transcription Factors
Source: PLoS Genet. 2016 Mar 28;12(3):e1005940. doi: 10.1371/journal.pgen.1005940 (PMC4809547; doi:10.1371/journal.pgen.1005940)

S1 Fig. Effects of depleting CHD1, CHD2 and CHD4 on promoter nucleosome organisation.

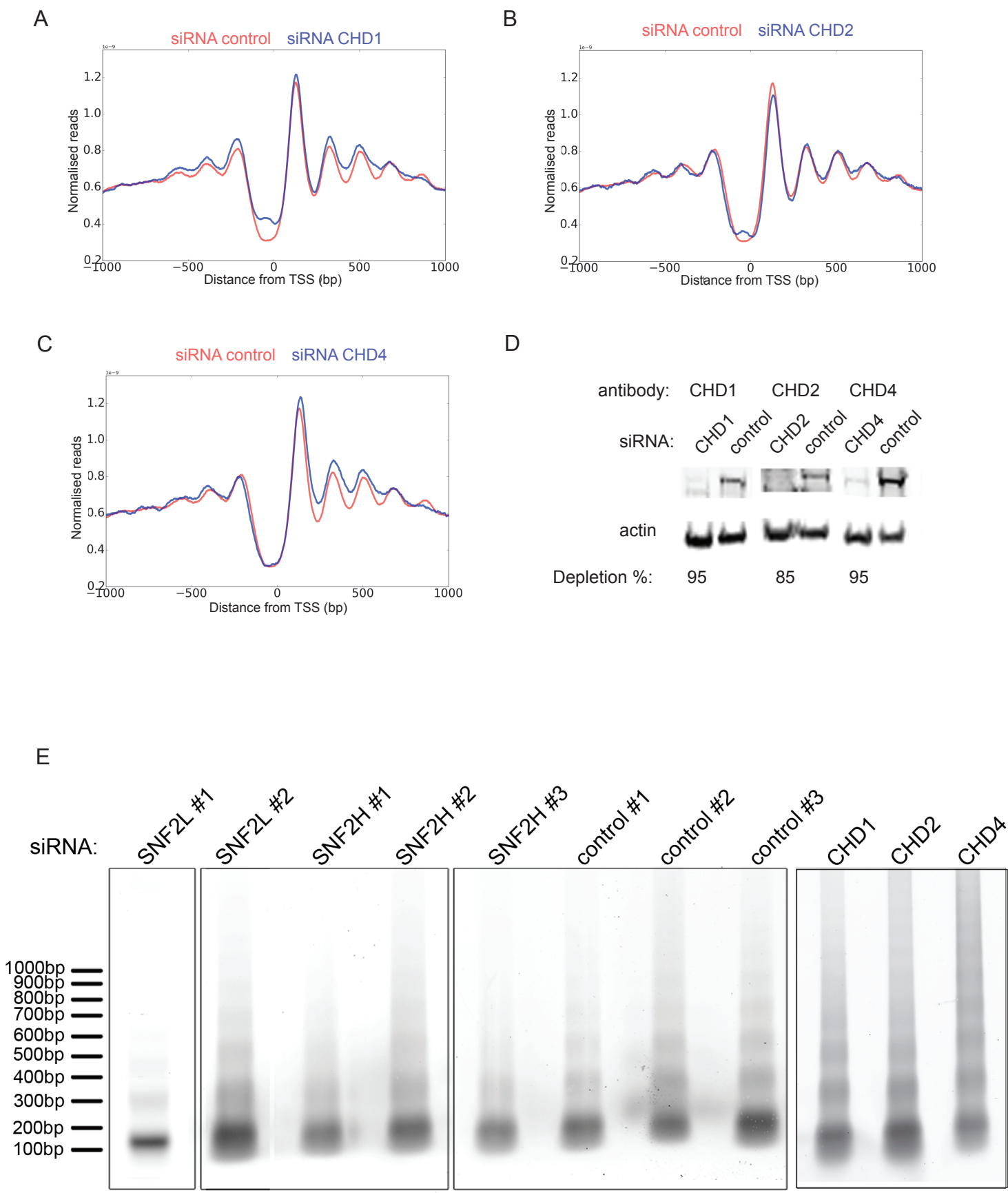

Supplement: S1 Fig — Nucleosomal reads aligned to the promoters of ubiquitously expressed promoters following depletion of CHD1 (A, blue), CHD2 (B, blue) and CHD4 (C, blue). The red graph depicts the control knock down using a scramble oligo (A, B, C, same in all panels). Depletion of these enzymes has minor effects on the organisation of nucleosomes at promoters. (D) Western blot showing siRNA knock down of CHD1, CHD2 and CHD4 compared to control knock down using a scramble oligo. Level of depletion was determined following normalization to a beta-actin loading control. (E) Isolation of mono nucleosomal DNA fragments following siRNA depletion. Agarose gel showing the DNA fragment length distribution obtained after higher levels of MNase digestion following depletion of the enzymes indicated. The mono nucleosome length species was gel purified and processed for sequencing. (PDF) [file pgen.1005940.s001.pdf]

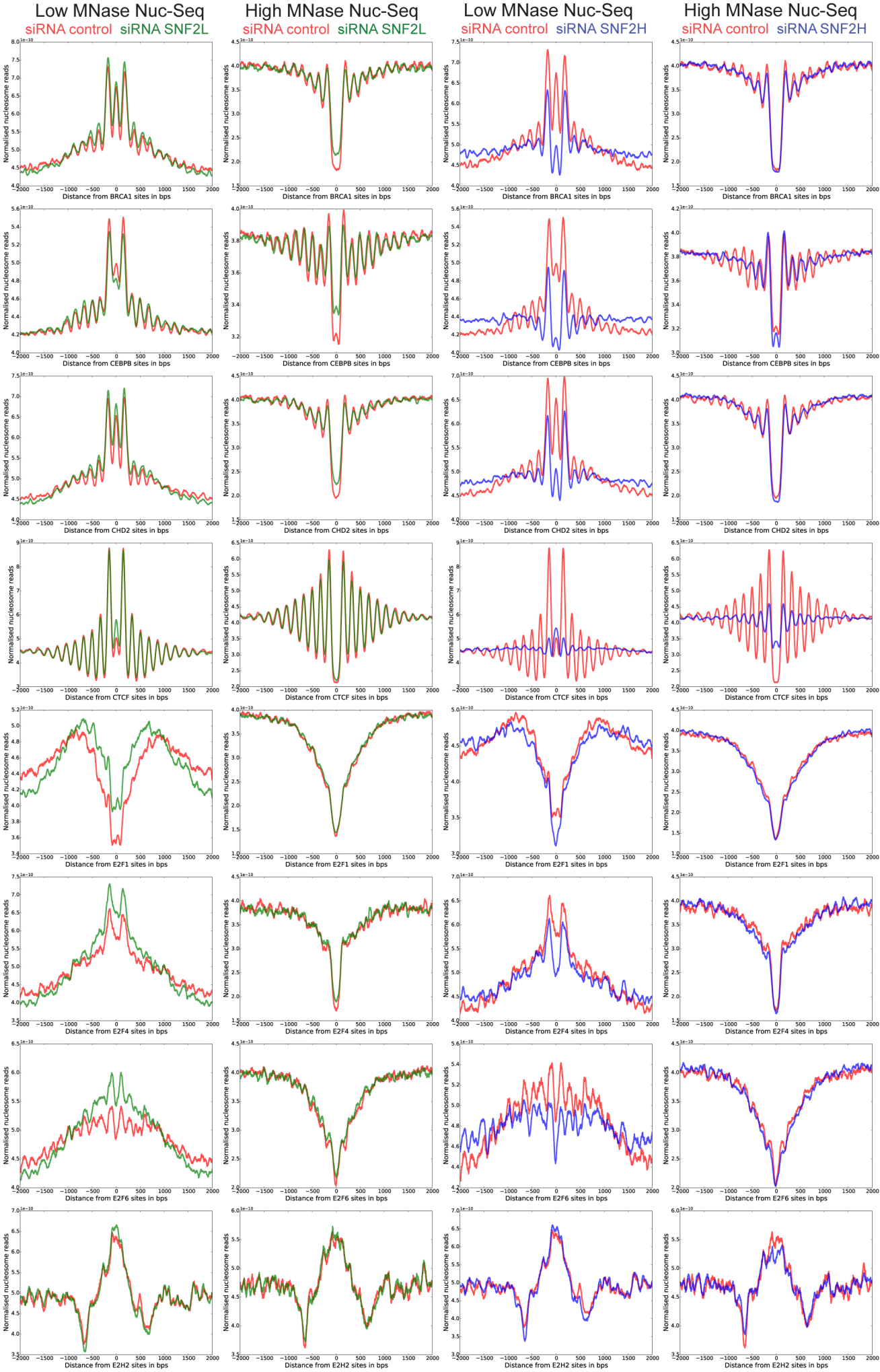

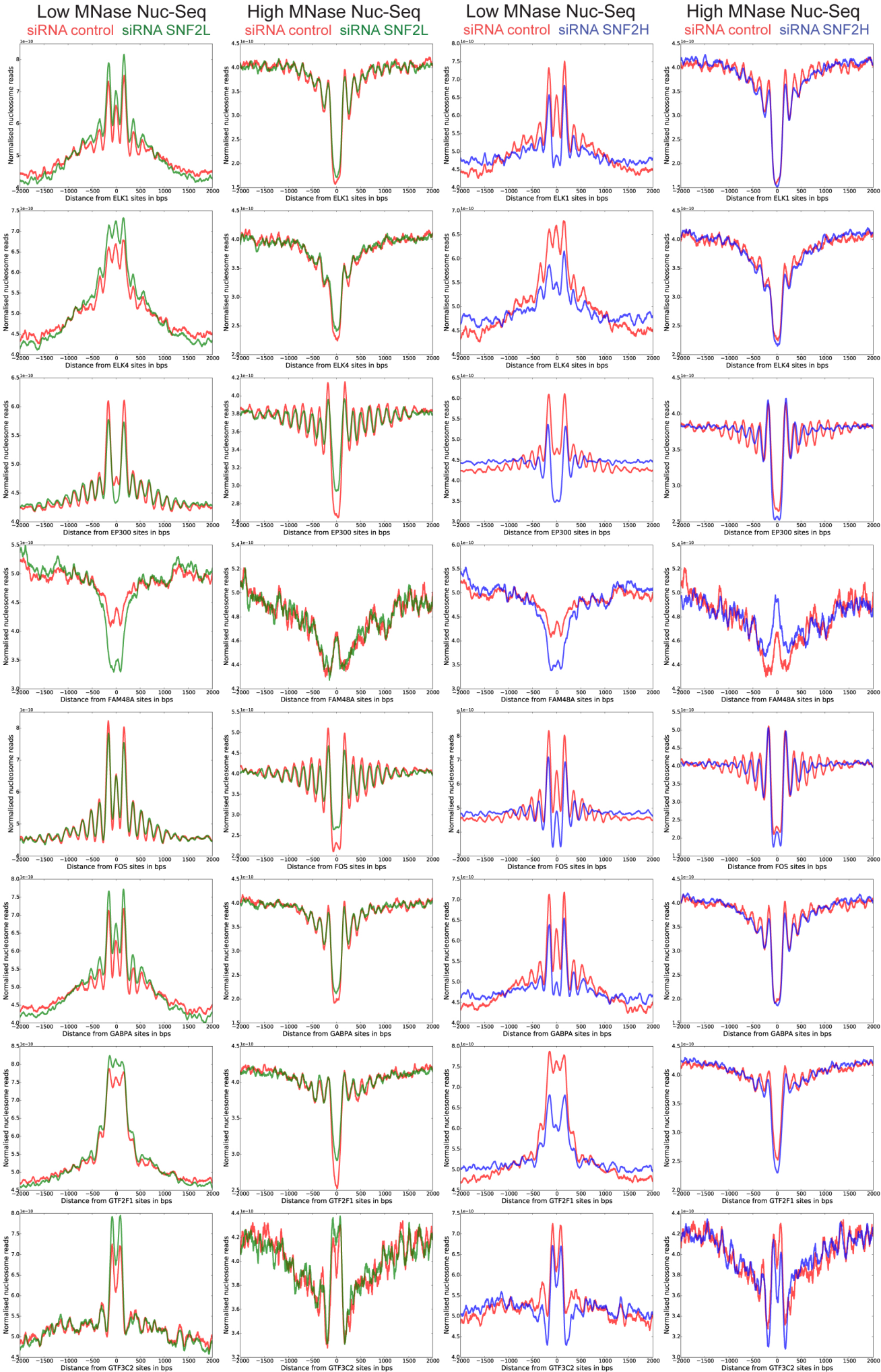

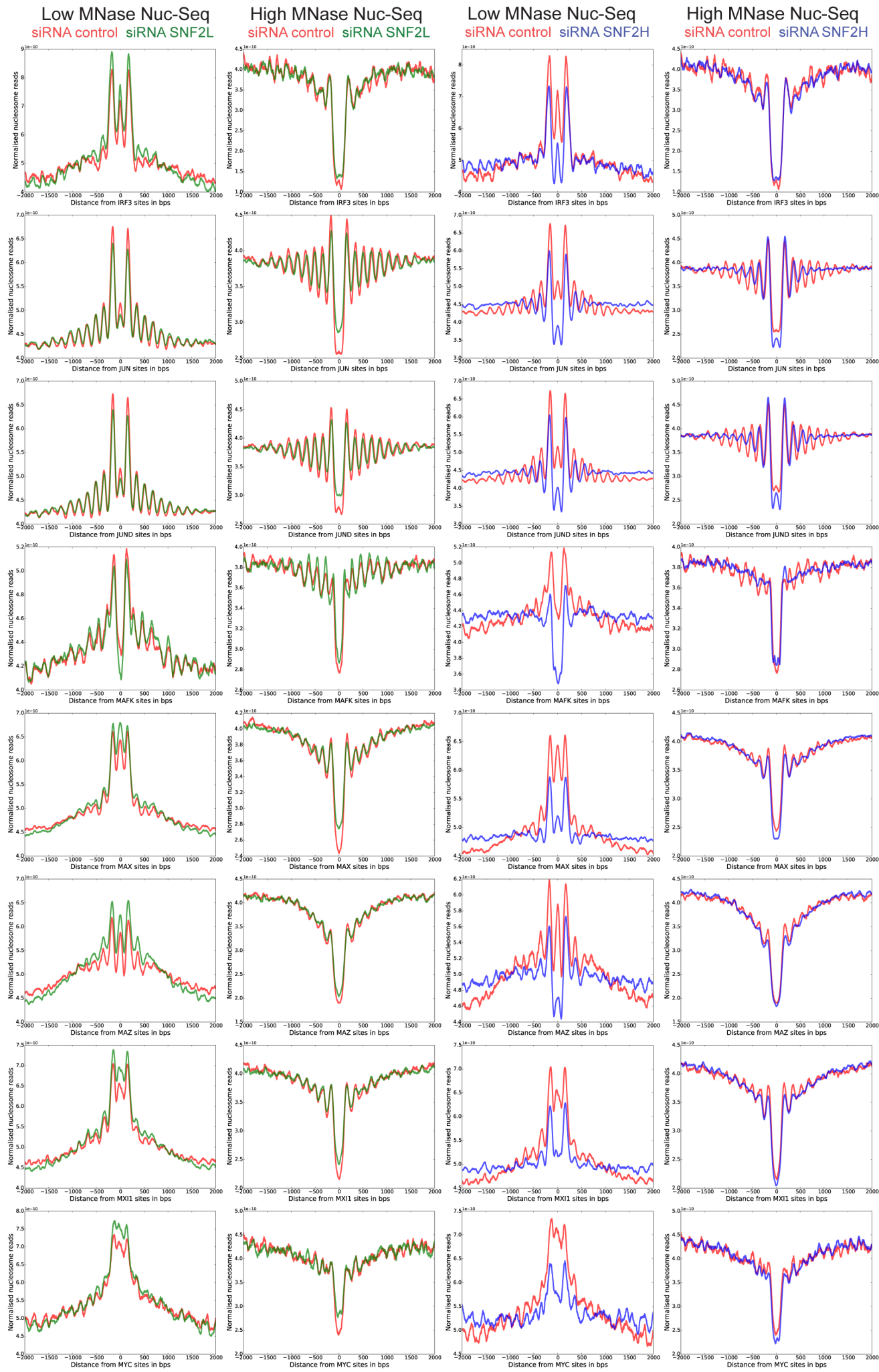

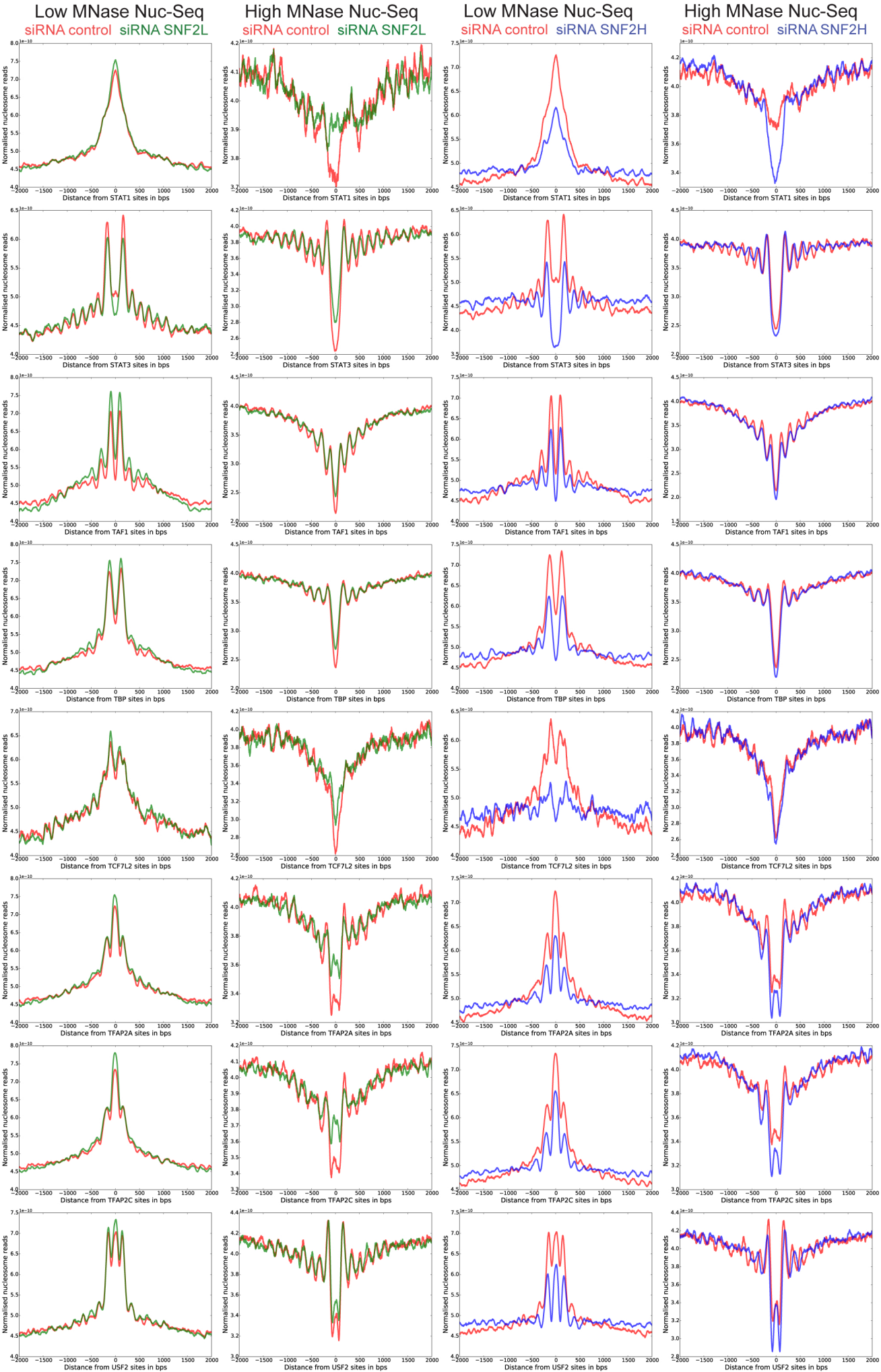

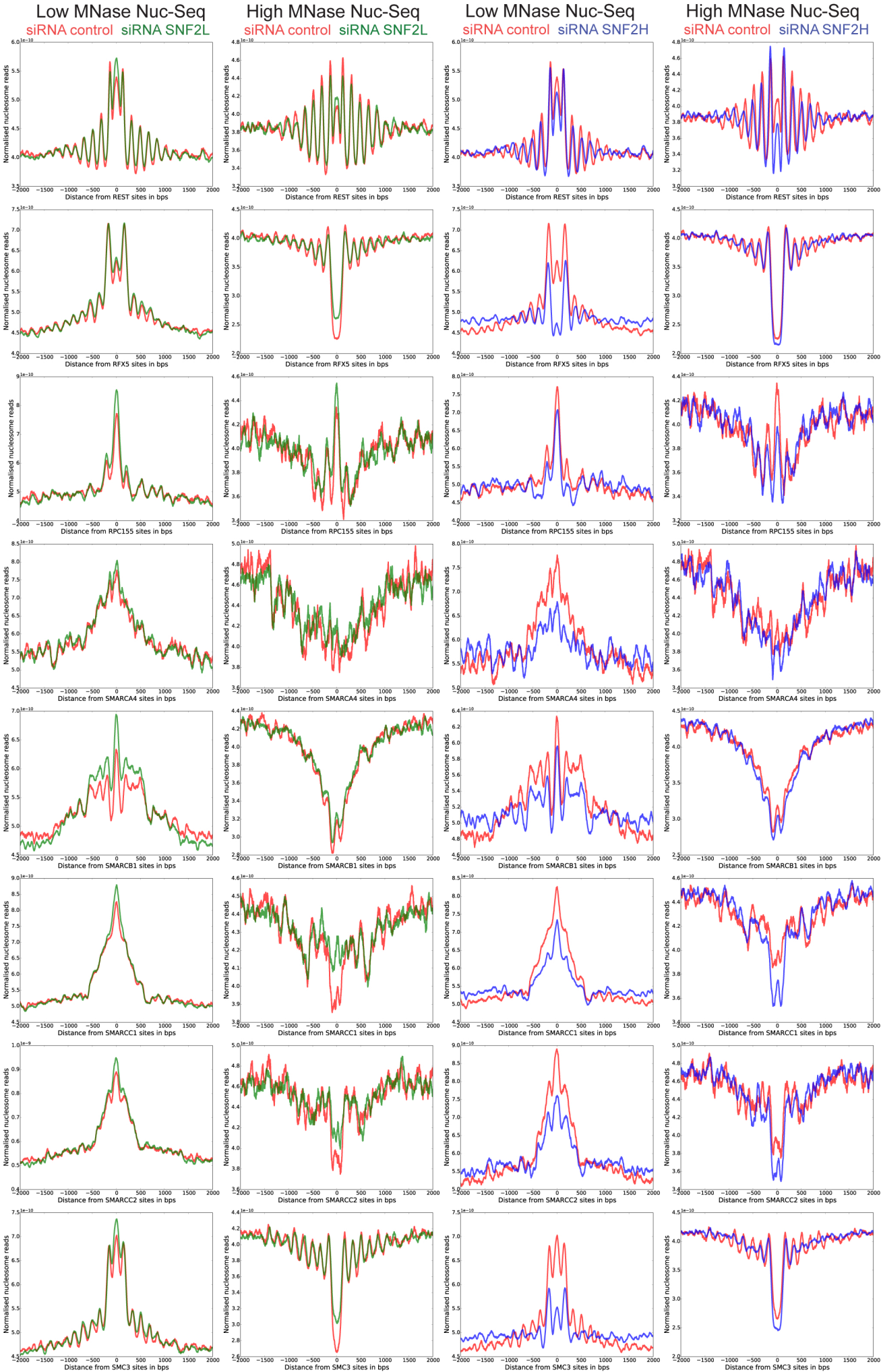

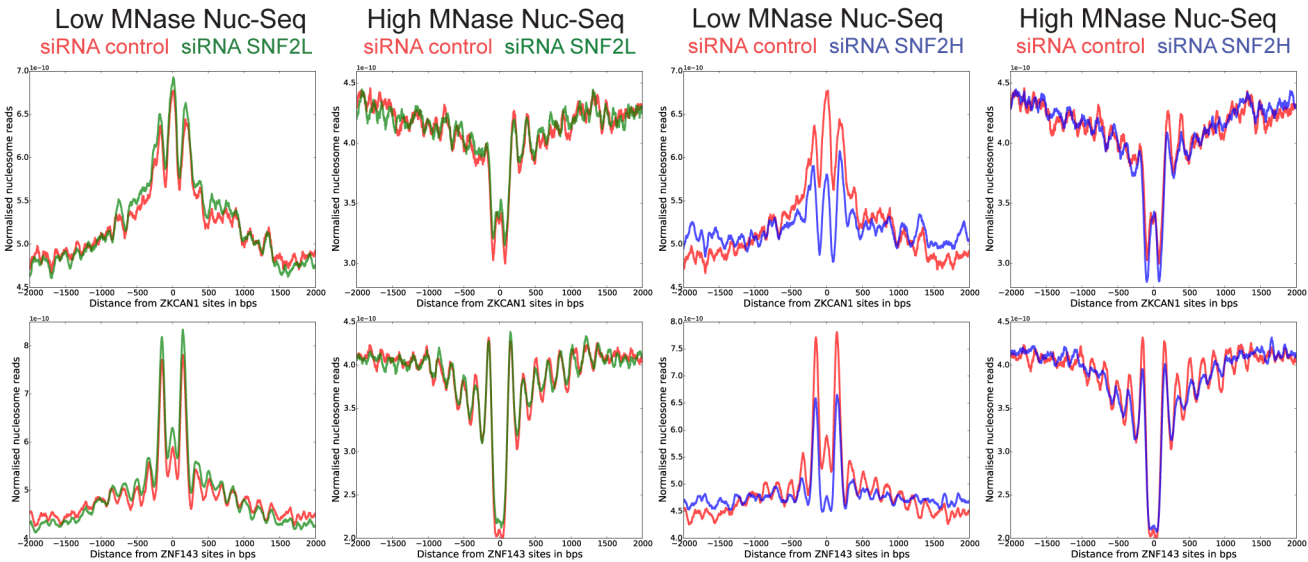

Supplement: S6 Fig — Nucleosome seq indicating the positioning of nucleosomes adjacent to 50 transcription factor binding sites after depletion of SNF2L and SNF2H after low (169 bp average nucleosome fragment length) or high MNase digestion (147 bp average nucleosome fragment length). Plots for all 50 factors for which ChIP data identifying at least 1000 bound sites in HeLa was available. The red plots are control knock downs using a scramble oligo while the green plots show SNF2L depletions and blue plots show SNF2H depletions. In all cases data was only taken from factor binding sites that do not have adjacent CTCF sites. (PDF) [file pgen.1005940.s006.pdf]

S7 Fig. SNF2H depletion does not change RAD21 occupancy at most factor binding sites.

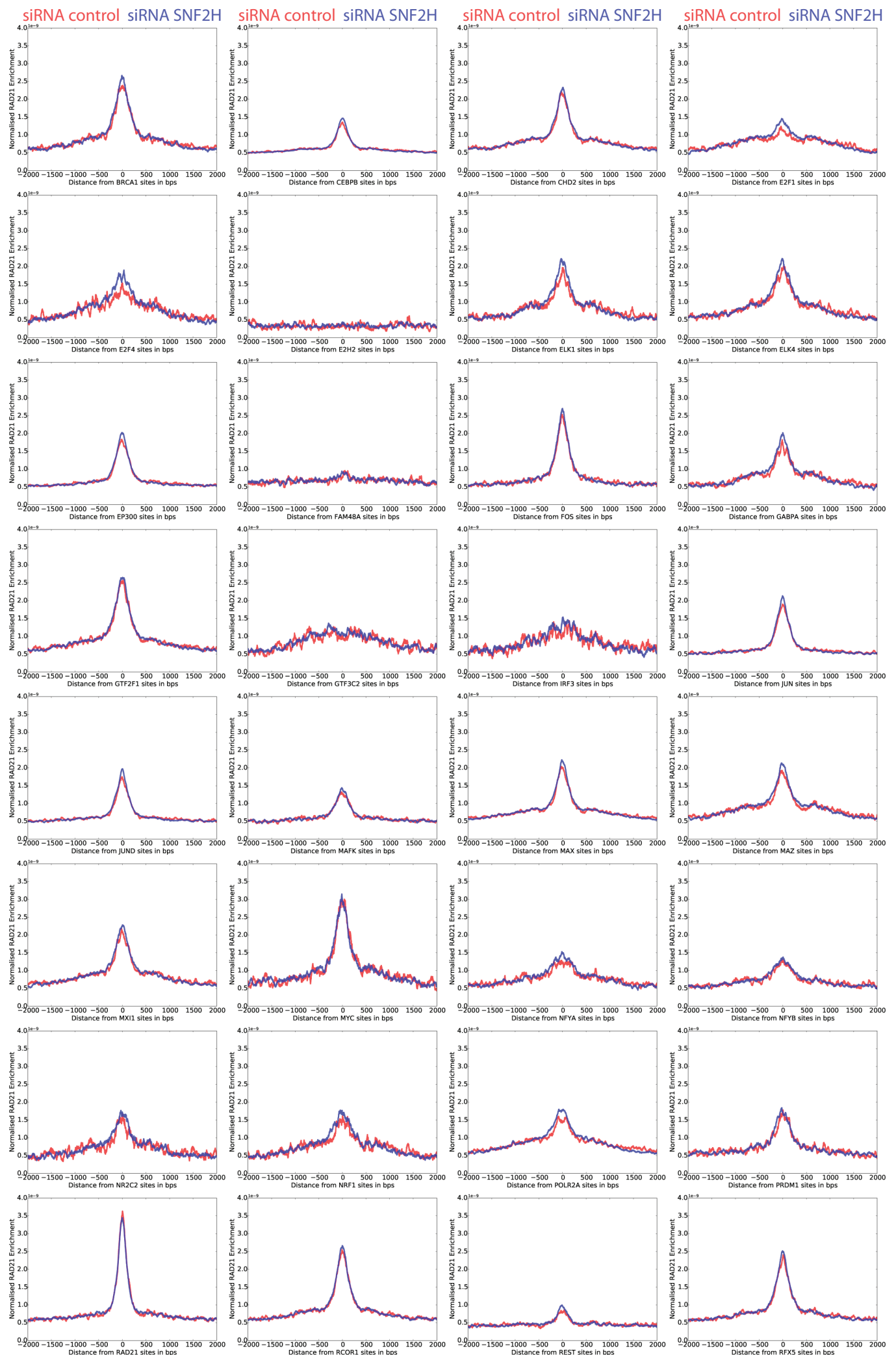

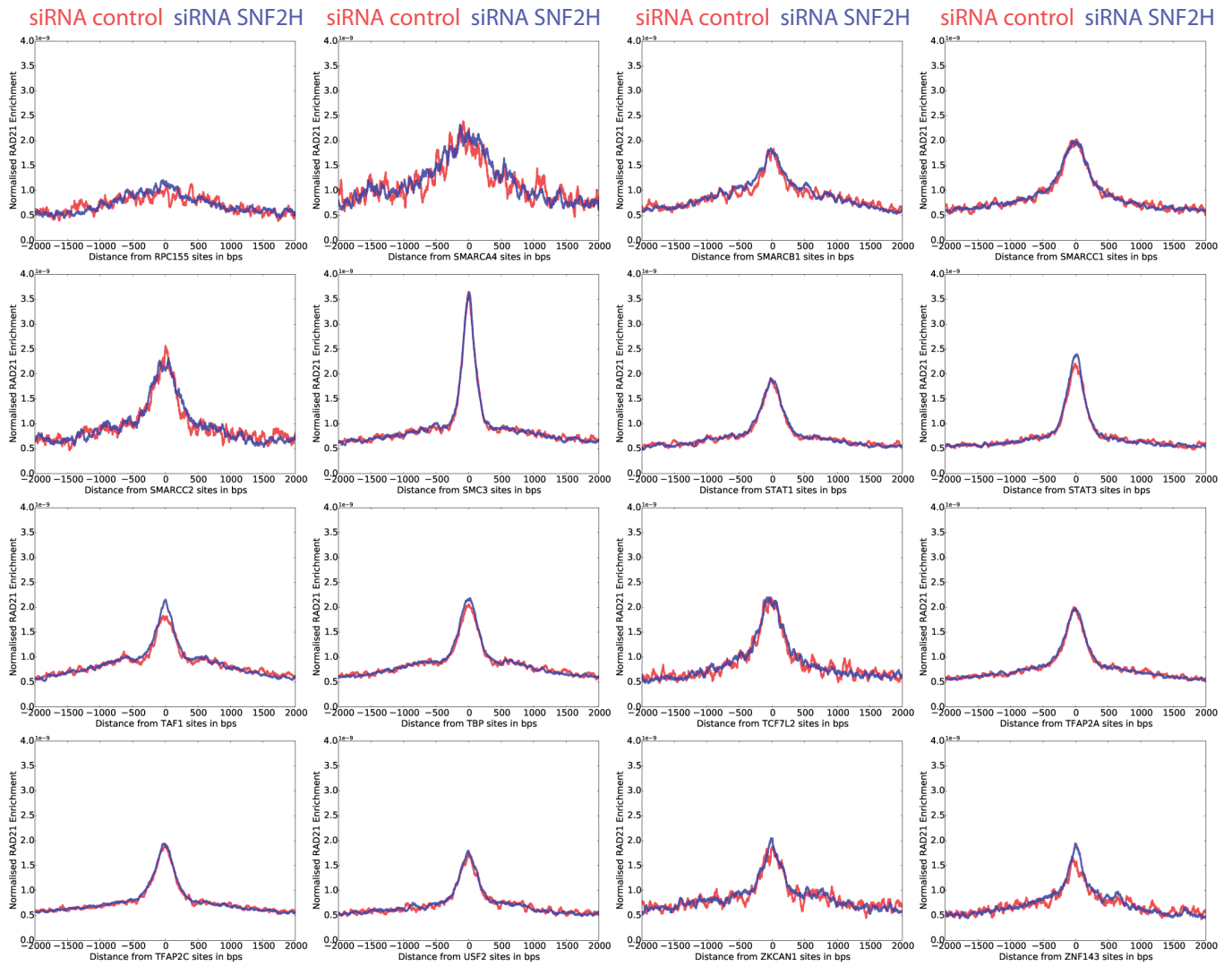

Supplement: S7 Fig — ChIP seq data for RAD21 ChIP after SNF2H depletion for 49 factor binding sites. RAD21 enrichment is shown after SNF2H depletion (blue) and in control cells (red). The removal of SNF2H has no effect on RAD21 enrichment at these factor binding sites which contrasts with the effect observed at CTCF sites shown in Fig 4B. (PDF) [file pgen.1005940.s007.pdf]
