# Supplementary material for: The Chromatin Remodelling Enzymes SNF2H and SNF2L Position Nucleosomes adjacent to CTCF and Other Transcription Factors
Source: PLoS Genet. 2016 Mar 28;12(3):e1005940. doi: 10.1371/journal.pgen.1005940 (PMC4809547; doi:10.1371/journal.pgen.1005940)

S2 Fig. Effects of depleting CHD1, CHD2 and CHD4 on nucleosome organisation adjacent to CTCF binding sites.

A

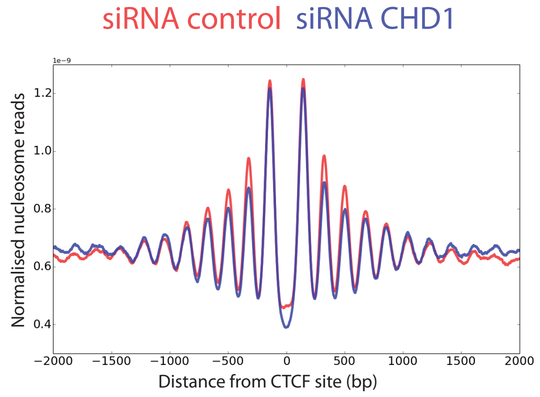

B

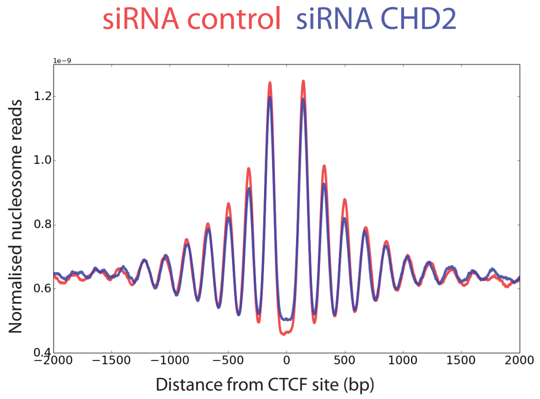

C

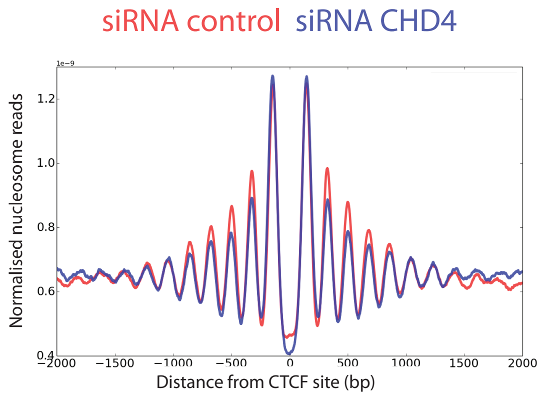

Supplement: S2 Fig — Nucleosome density plots of sequenced mono nucleosomal DNA fragments after depletion of CHD1 (A), CHD2 (B), and CHD4 (C) proteins aligned to CTCF binding sites. Knock down of the indicated proteins results in relatively subtle changes to the nucleosomal profile. (PDF) [file pgen.1005940.s002.pdf]
