# Supplementary material for: The Chromatin Remodelling Enzymes SNF2H and SNF2L Position Nucleosomes adjacent to CTCF and Other Transcription Factors
Source: PLoS Genet. 2016 Mar 28;12(3):e1005940. doi: 10.1371/journal.pgen.1005940 (PMC4809547; doi:10.1371/journal.pgen.1005940)

S3 Fig. Depletion of subunits of SNF2H and SNF2L containing complexes has minor effects on nucleosome organisation adjacent to CTCF binding sites.

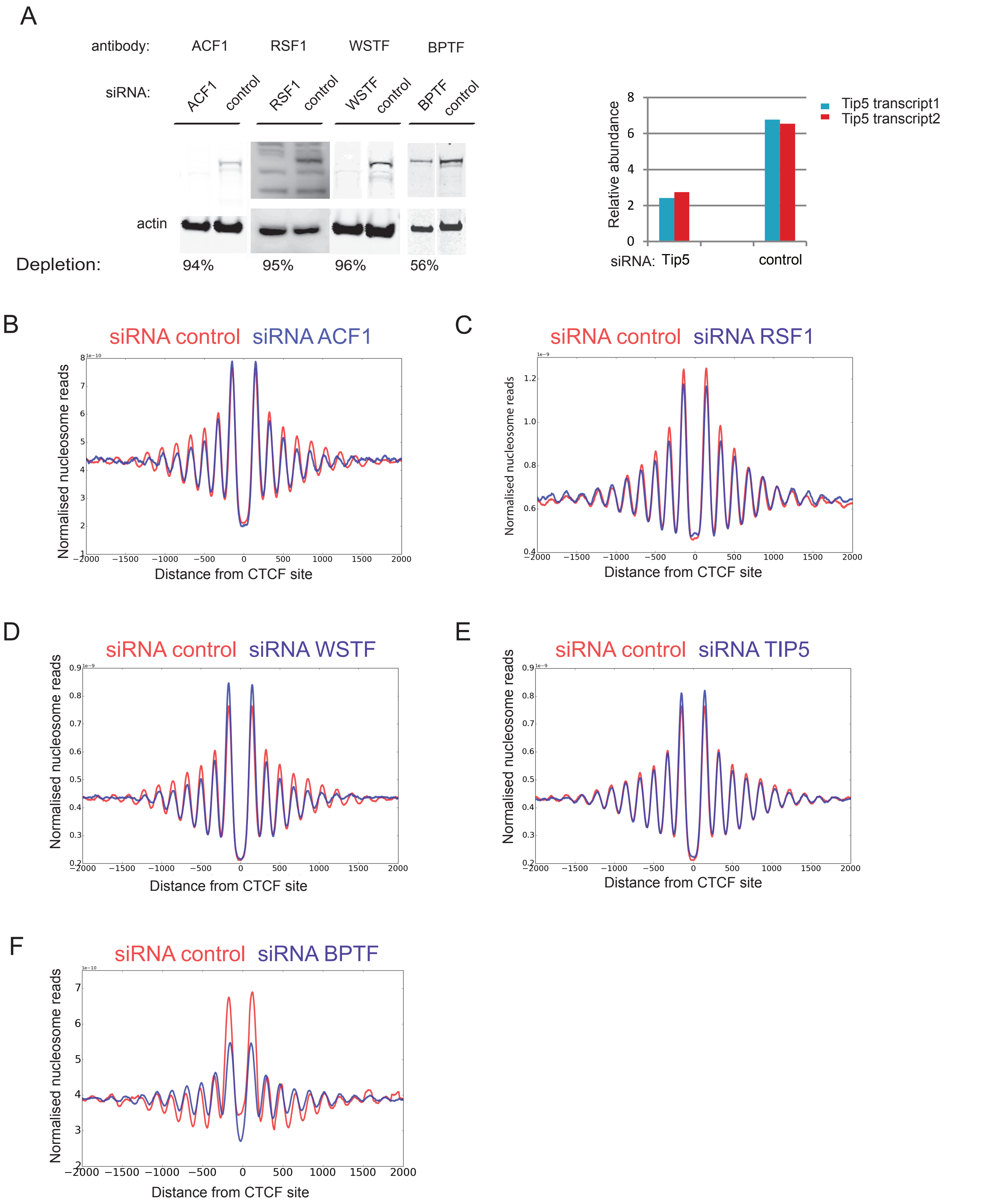

Supplement: S3 Fig — (A)Western blot showing siRNA knock down of ACF1, RSF1, WSTF and BPTF proteins compared to control knock down using scramble oligo. Level of depletion was determined using infrared fluorescence normalised to a beta-actin loading control. Antibodies used as indicated. Due to the lack of a functional antibody, TIP5 depletion of 68% was measured using real time qPCR using two different amplicons. (B-F) Nucleosome density plots of sequenced mono nucleosomal DNA after depletion of SNF2H complex subunits ACF1 (B), RSF1 (C), WSTF (D) and TIP5 (E) proteins and NURF complex subunit BPTF (F) mapped to CTCF binding sites. Knock down of the SNF2H complex subunits result only in minor changes to the distribution of nucleosomal reads while the knock down of BPTF shows a stronger effect on nucleosome occupancy at CTCF binding sites. (PDF) [file pgen.1005940.s003.pdf]
