# Supplementary material for: The Chromatin Remodelling Enzymes SNF2H and SNF2L Position Nucleosomes adjacent to CTCF and Other Transcription Factors
Source: PLoS Genet. 2016 Mar 28;12(3):e1005940. doi: 10.1371/journal.pgen.1005940 (PMC4809547; doi:10.1371/journal.pgen.1005940)
Supplement: S4 Fig — The removal of coincident CTCF sites greatly reduces CTCF occupancy determined by ChIP at the binding sites for a disparate range of transcription factors. CTCF ChIP seq was plotted at 50 transcription factor binding sites including all sites (red) or sites with CTCF sites within 500bp removed (blue). Nuc seq at 50 transcription factor binding sites was plotted with (red) and without (blue) CTCF sites within 500bp. For many different transcription factors adjacent CTCF binding sites contribute to the nucleosome organisation observed when averaging all sites. (PDF) [file pgen.1005940.s004.pdf]

S4 Fig. CTCF sites interfere with the nucleosome organization at transcription factor binding sites.

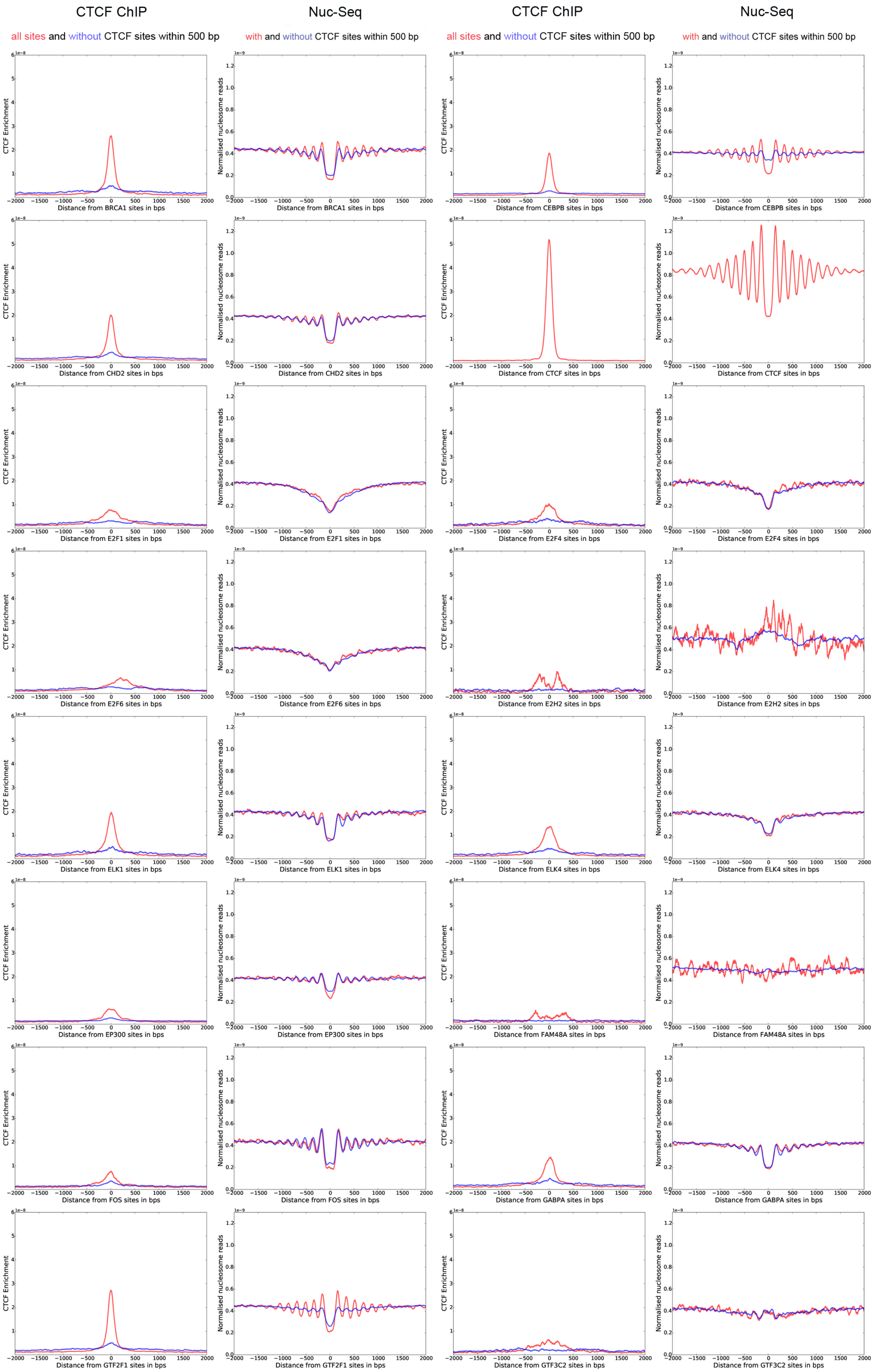

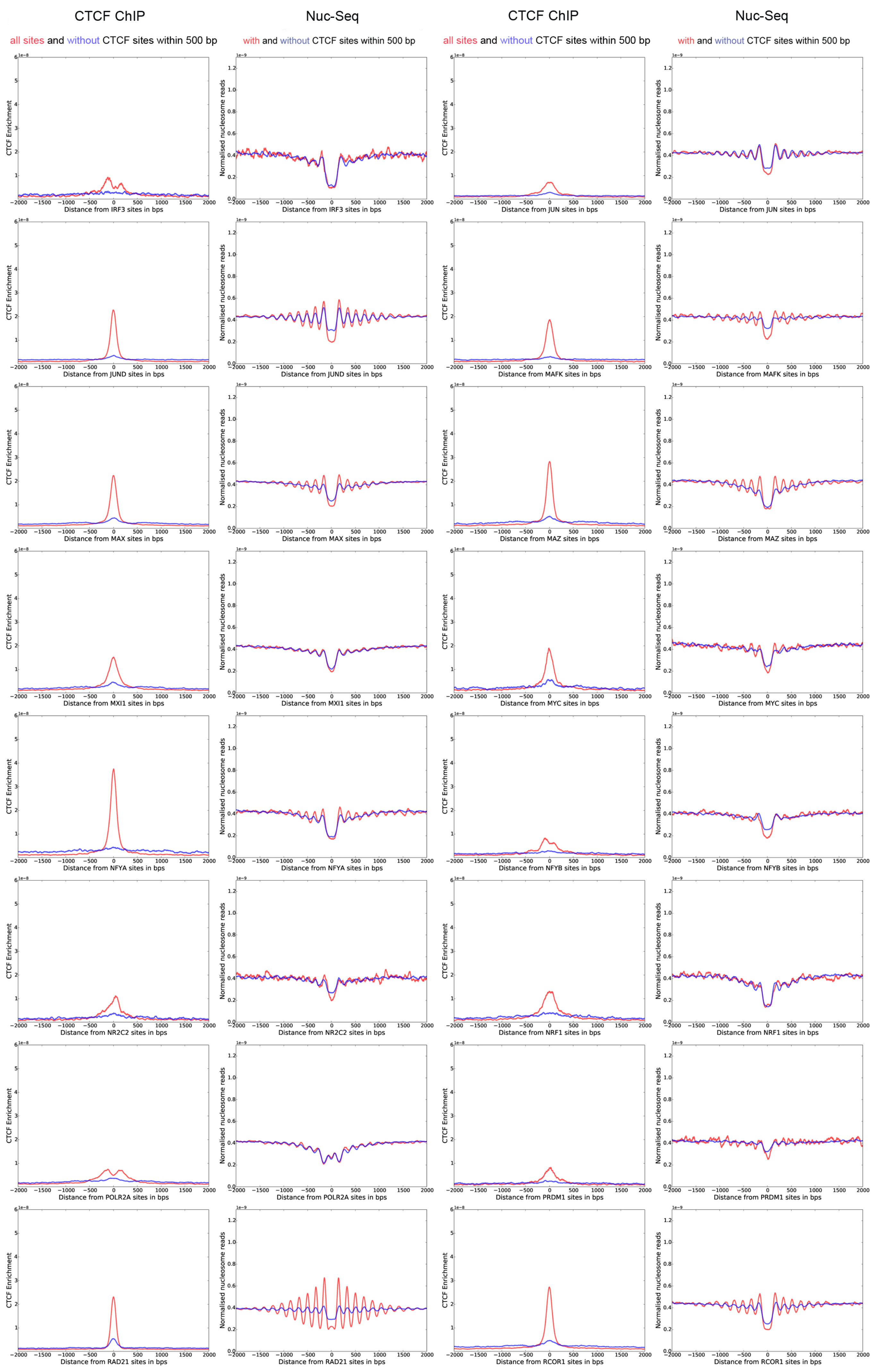
CTCF ChIP
all sites and without CTCF sites within 500 bp
Nuc-Seq
with and without CTCF sites within 500 bp
CTCF ChIP
all sites and without CTCF sites within 500 bp
Nuc-Seq
with and without CTCF sites within 500 bp
CTCF Enrichment
Normalised nucleosome reads
1e-8
1e-9
Distance from IRF3 sites in bps
Distance from JUN sites in bps
Distance from JUND sites in bps
Distance from MAFK sites in bps
Distance from MAX sites in bps
Distance from MAZ sites in bps
Distance from MXI1 sites in bps
Distance from MYC sites in bps
Distance from NFYA sites in bps
Distance from NFYB sites in bps
Distance from NR2C2 sites in bps
Distance from NRF1 sites in bps
Distance from POLR2A sites in bps
Distance from PRDM1 sites in bps
Distance from RAD21 sites in bps
Distance from RCOR1 sites in bps

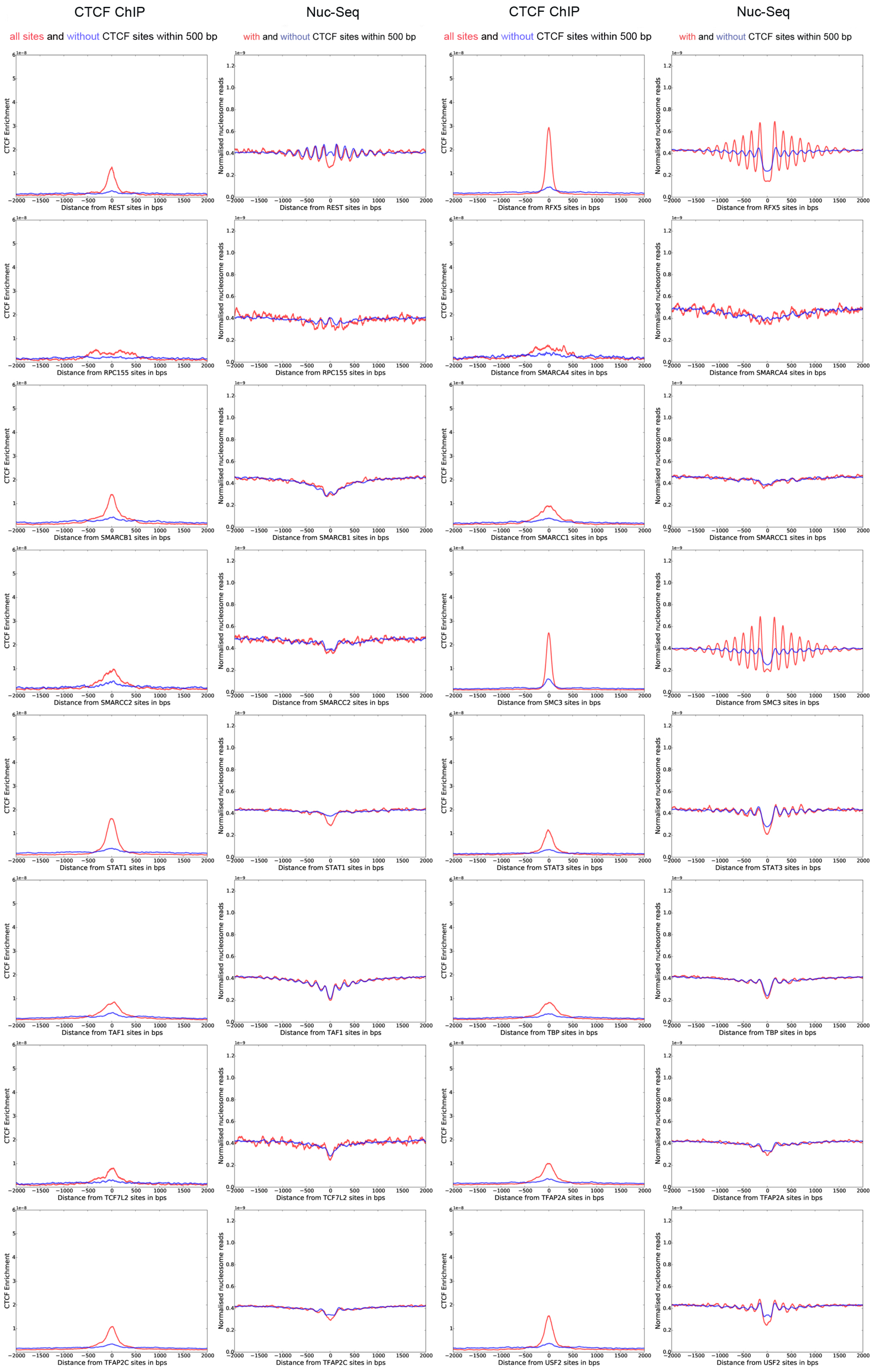
CTCF ChIP
all sites and without CTCF sites within 500 bp
Nuc-Seq
with and without CTCF sites within 500 bp
CTCF ChIP
all sites and without CTCF sites within 500 bp
Nuc-Seq
with and without CTCF sites within 500 bp
CTCF Enrichment
Normalised nucleosome reads
Distance from REST sites in bps
Distance from RFX5 sites in bps
Distance from RPC155 sites in bps
Distance from SMARCA4 sites in bps
Distance from SMARCB1 sites in bps
Distance from SMARCC1 sites in bps
Distance from SMARCC2 sites in bps
Distance from SMC3 sites in bps
Distance from STAT1 sites in bps
Distance from STAT3 sites in bps
Distance from TAF1 sites in bps
Distance from TBP sites in bps
Distance from TCF7L2 sites in bps
Distance from TFAP2A sites in bps
Distance from TFAP2C sites in bps
Distance from USF2 sites in bps

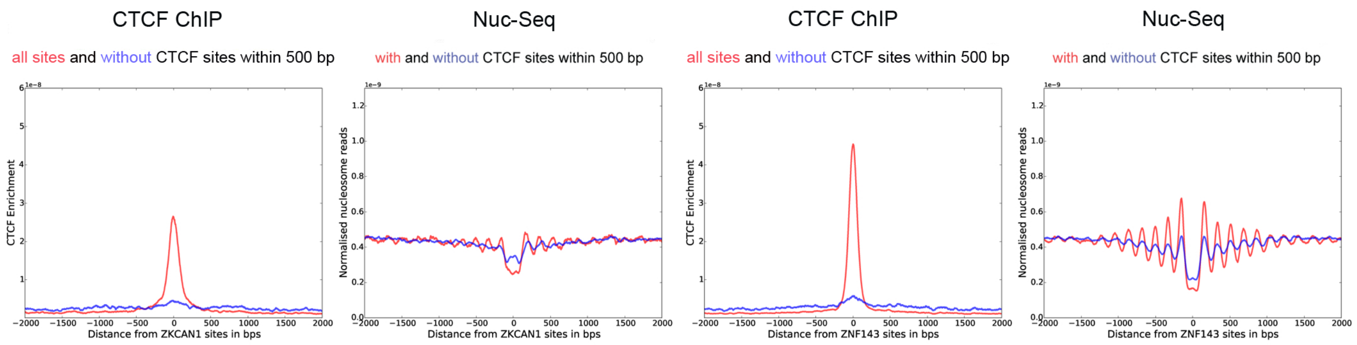
CTCF ChIP
all sites and without CTCF sites within 500 bp
CTCF Enrichment
Distance from ZKCAN1 sites in bps
Nuc-Seq
with and without CTCF sites within 500 bp
Normalised nucleosome reads
Distance from ZKCAN1 sites in bps
CTCF ChIP
all sites and without CTCF sites within 500 bp
CTCF Enrichment
Distance from ZNF143 sites in bps
Nuc-Seq
with and without CTCF sites within 500 bp
Normalised nucleosome reads
Distance from ZNF143 sites in bps
