# Supplementary material for: The Chromatin Remodelling Enzymes SNF2H and SNF2L Position Nucleosomes adjacent to CTCF and Other Transcription Factors
Source: PLoS Genet. 2016 Mar 28;12(3):e1005940. doi: 10.1371/journal.pgen.1005940 (PMC4809547; doi:10.1371/journal.pgen.1005940)
Supplement: S5 Fig — ChIP seq data for SNF2H and BPTF (first and third panel) at 50 transcription factor binding sites for which at least 1000 bound sites in HeLa cells were identified previously. Second and fourth panel show RAD21 ChIP seq data at factor binding sites plotted with (red) and without (blue) CTCF sites within 500 bp. (PDF) [file pgen.1005940.s005.pdf]

S5 Fig. Enrichment of chromatin remodelling enzymes and cohesin at the binding sites for different transcription factors.

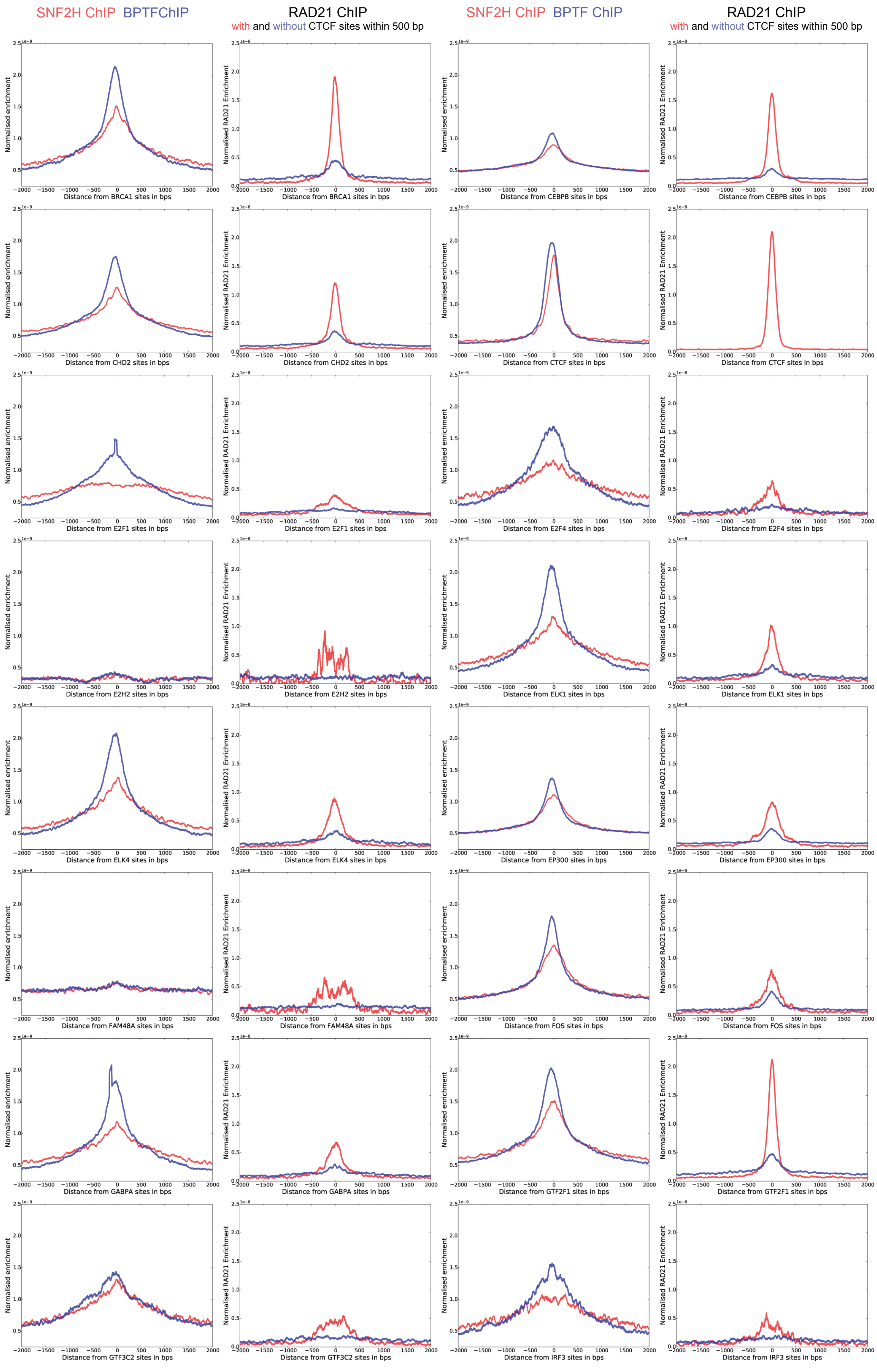

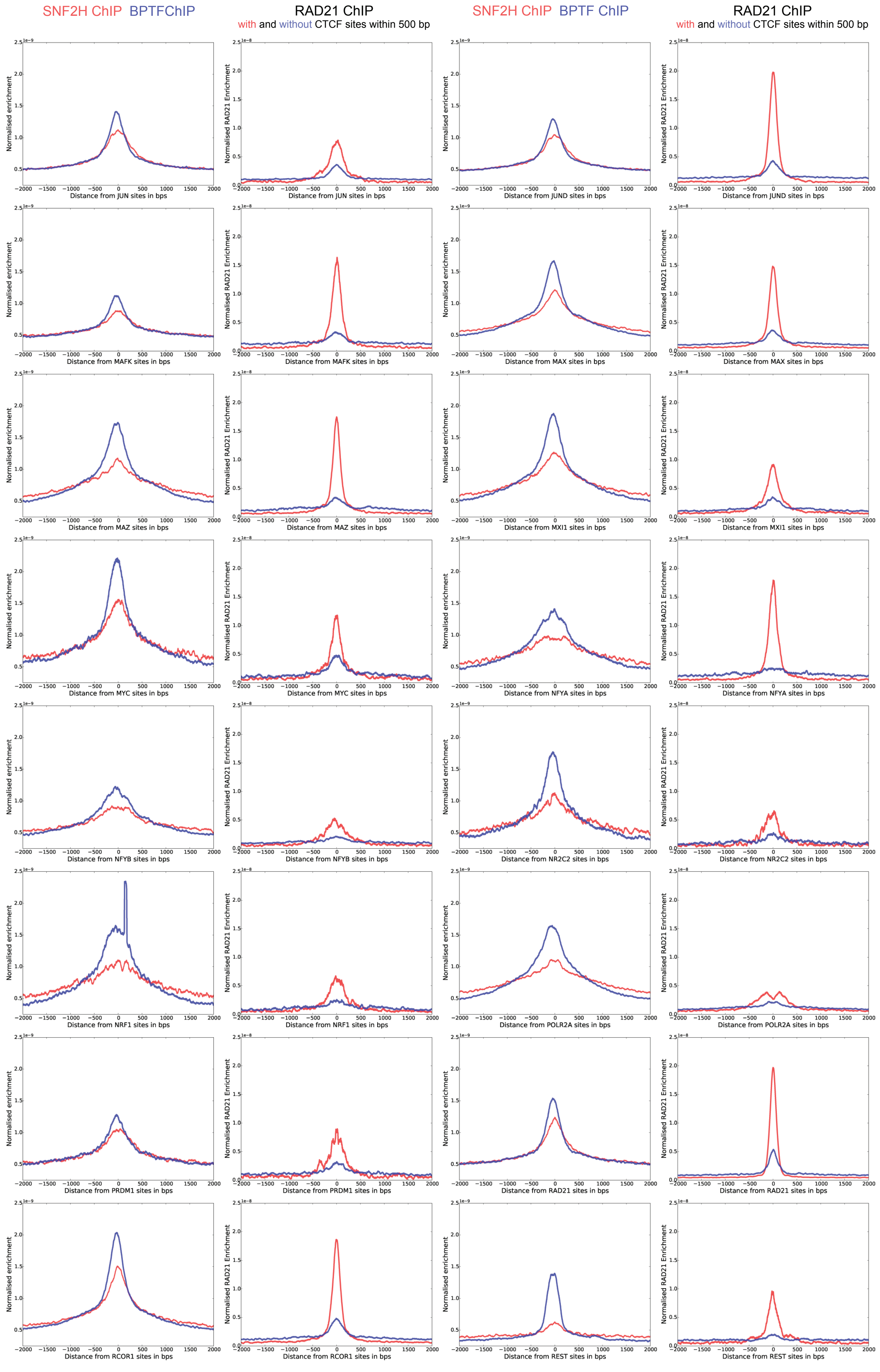
SNF2H ChIP BPTFChIP
RAD21 ChIP
with and without CTCF sites within 500 bp
SNF2H ChIP BPTF ChIP
RAD21 ChIP
with and without CTCF sites within 500 bp
Normalised enrichment
Normalised RAD21 Enrichment
Distance from JUN sites in bps
Distance from JUND sites in bps
Distance from MAFK sites in bps
Distance from MAX sites in bps
Distance from MAZ sites in bps
Distance from MXI1 sites in bps
Distance from MYC sites in bps
Distance from NFYA sites in bps
Distance from NFYB sites in bps
Distance from NR2C2 sites in bps
Distance from NRF1 sites in bps
Distance from POLR2A sites in bps
Distance from PRDM1 sites in bps
Distance from RAD21 sites in bps
Distance from RCOR1 sites in bps
Distance from REST sites in bps

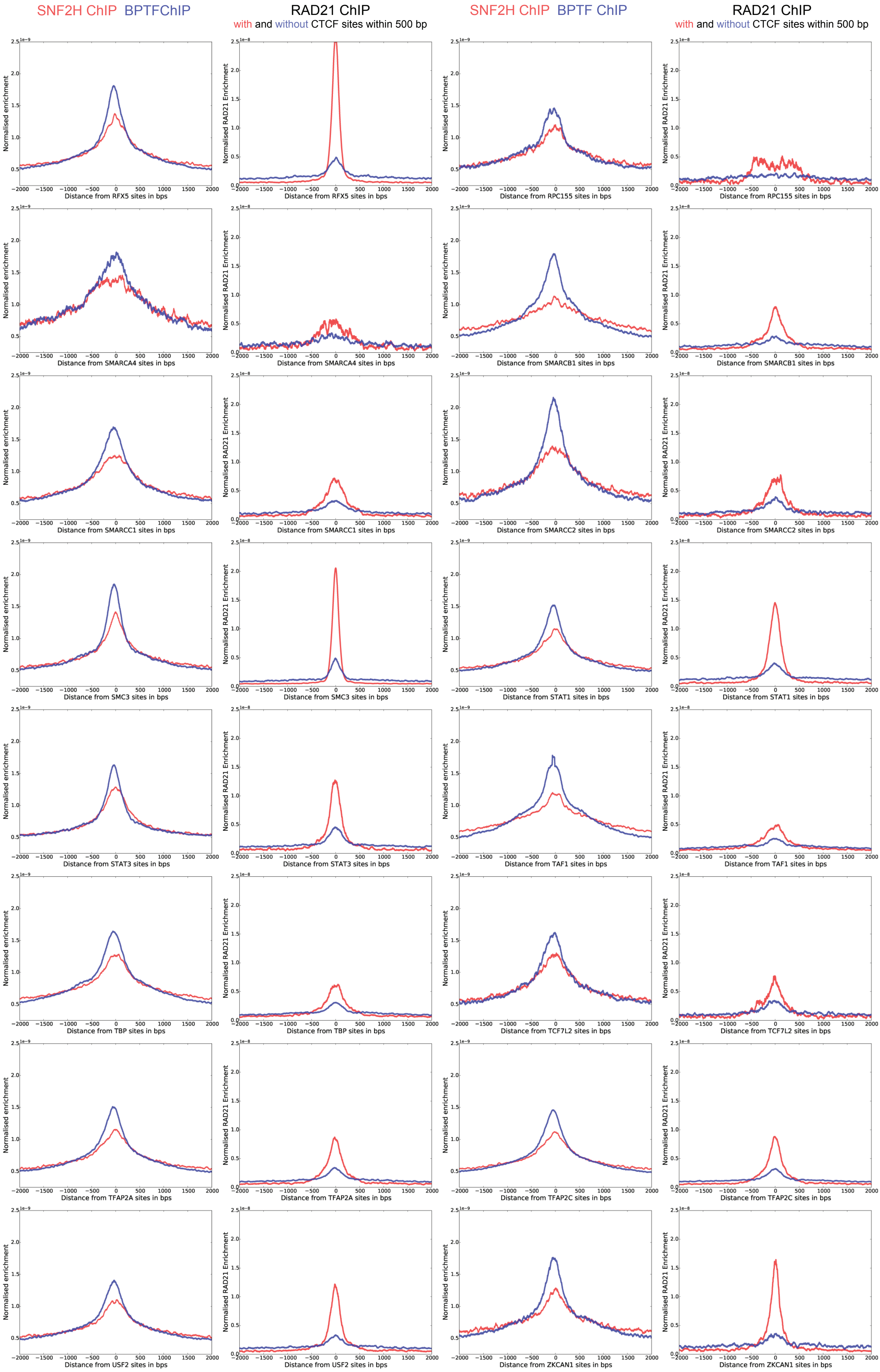
SNF2H ChIP BPTFChIP
RAD21 ChIP
with and without CTCF sites within 500 bp
SNF2H ChIP BPTF ChIP
RAD21 ChIP
with and without CTCF sites within 500 bp
Normalised enrichment
Normalised RAD21 Enrichment
Distance from RFX5 sites in bps
Distance from RFX5 sites in bps
Distance from RPC155 sites in bps
Distance from RPC155 sites in bps
Distance from SMARCA4 sites in bps
Distance from SMARCA4 sites in bps
Distance from SMARCB1 sites in bps
Distance from SMARCB1 sites in bps
Distance from SMARCC1 sites in bps
Distance from SMARCC1 sites in bps
Distance from SMARCC2 sites in bps
Distance from SMARCC2 sites in bps
Distance from SMC3 sites in bps
Distance from SMC3 sites in bps
Distance from STAT1 sites in bps
Distance from STAT1 sites in bps
Distance from STAT3 sites in bps
Distance from STAT3 sites in bps
Distance from TAF1 sites in bps
Distance from TAF1 sites in bps
Distance from TBP sites in bps
Distance from TBP sites in bps
Distance from TCF7L2 sites in bps
Distance from TCF7L2 sites in bps
Distance from TFAP2A sites in bps
Distance from TFAP2A sites in bps
Distance from TFAP2C sites in bps
Distance from TFAP2C sites in bps
Distance from USF2 sites in bps
Distance from USF2 sites in bps
Distance from ZKCAN1 sites in bps
Distance from ZKCAN1 sites in bps

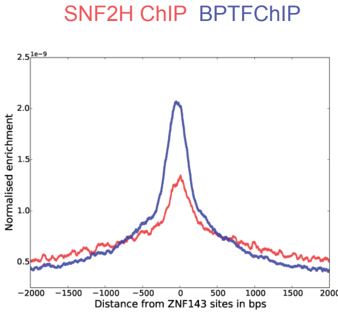
SNF2H ChIP BPTFChIP
Normalised enrichment
1e−9
2.5
2.0
1.5
1.0
0.5
−2000 −1500 −1000 −500 0 500 1000 1500 2000
Distance from ZNF143 sites in bps

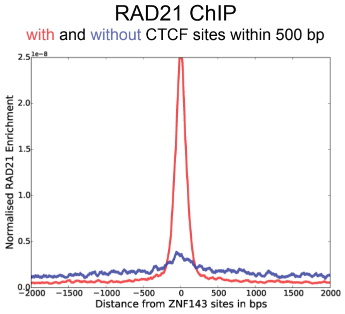
RAD21 ChIP
with and without CTCF sites within 500 bp
Normalised RAD21 Enrichment
1e−8
2.5
2.0
1.5
1.0
0.5
0.0
−2000 −1500 −1000 −500 0 500 1000 1500 2000
Distance from ZNF143 sites in bps
