# Supplementary material for: The Chromatin Remodelling Enzymes SNF2H and SNF2L Position Nucleosomes adjacent to CTCF and Other Transcription Factors
Source: PLoS Genet. 2016 Mar 28;12(3):e1005940. doi: 10.1371/journal.pgen.1005940 (PMC4809547; doi:10.1371/journal.pgen.1005940)
Supplement: S1 Table — The most abundant read length, read depth and anticipated coverage are indicated for each sequence dataset generated for this study. Also included is a description of which datasets are plotted in each figure. (PDF) [file pgen.1005940.s008.pdf]

Sample	Fragment Length (bp)	read_depth	Coverage
siACF1_rep1	162	90244092	4.0
siACF1_rep2	169	224383130	12
siBPTF1_rep1	182	65319132	3.0
siBPTF1_rep2	165	30462118	1.02
siCHD1_rep1	156	67789368	4.52
siCHD1_rep2	165	42948831	2.86
siCHD2_rep1	158	30548038	2.04
siCHD2_rep2	156	42799783	2.85
siCHD4_rep1	175	32430629	2.16
siCHD4_rep2	163	34716424	2.31
siCTCF_rep1	158	30636284	2.04
siCTCF_rep2	167	40564258	2.70
siMTA2_rep1	162	68992272	4.60
siMTA2_rep2	163	37728046	2.52
siRSF1_rep1	159	38025399	2.54
siRSF1_rep2	165	75016828	5.00
siScr_rep1	152	92948969	6.20
siScr_rep2	150	41563088	2.77
siScr_rep3	165	31579576	1.02
siScr_rep4	169	262786584	14
siSNF2H_rep1	166	59782258	3.99
siSNF2H_rep2	142	36562135	2.44
siSNF2L_rep1	154	76379632	5.09
siSNF2L_rep2	164	57884732	3.86
siTIP5_rep1	158	103696848	6.91
siTIP5_rep2	170	37608848	2.51
siWSTF_rep1	159	68099204	4.54
siWSTF_rep2	171	36588148	2.44
**High Read Depth Data Sets**			
siScr_Low-Mnase_1	154	321425328	17
siScr_Low-Mnase_2	174	293039656	16
siScr_Low-Mnase_3	174	258400534	14
siScr_High-Mnase_1	150	265451086	19
siScr_High-Mnase_2	149	350271356	11
siScr_High-Mnase_3	151	213500428	18
siSNF2H_Low-Mnase_1	169	307067954	16
siSNF2H_Low-Mnase_2	172	311323692	17
siSNF2H_Low-Mnase_3	169	304385642	16
siSNF2H_High-Mnase_1	139	344581322	18
siSNF2H_High-Mnase_2	145	279702968	15
siSNF2H_High-Mnase_3	149	258470560	14
siSNF2L_Low-Mnase_1	169	320920510	17
siSNF2L_Low-Mnase_2	167	331801034	18
siSNF2L_Low-Mnase_3	173	278119682	15

siSNF2L_High-Mnase_1	148	348031584	19
siSNF2L_High-Mnase_2	148	250531556	13

Datasets used in Figures

Figure	Panel	Control	Sample
Figure1	B	siScr_High-Mnase_1-3	siSNF2H_High-Mnase_1-3
			siSNF2L_High-Mnase_1-2
	C	siScr_Low-Mnase_1 - 3	siSNF2H_Low-Mnase_1 - 3
			siSNF2L_Low-Mnase_1 -3
Figure2	A	siScr_rep2	siCTCF_rep2
	B	siScr_High-Mnase_1-3	siSNF2H_Low-Mnase_1 - 3
	C	siScr_High-Mnase_1-3	siSNF2L_Low-Mnase_1 -3
Figure3	A	siScr_SNF2H_rep1	siCTCF_SNF2H_rep1
	B	siScr_CTCF_rep2	siSNF2H_CTCF_rep2
Figure4	A	siScr_RAD21_rep2	siCTCF_RAD21_rep2
	B	siScr_RAD21_rep2	siSNF2H_RAD21_rep2
	C	siScr_SNF2H_rep2	siRAD21_SNF2H_rep2
	D	siScr1_rep1	siRAD21_rep1
Figure5	A,B	siScr_CTCF_rep1	
	C-G	siScr_High-Mnase_1-3	
	H,I	siScr_RAD21_rep1	siCTCF_RAD21_rep1
figure6	A,C,E,G	siScr_High-Mnase_1-3	siSNF2L_High-Mnase_1-2
	B,D,F,H	siScr_High-Mnase_1-3	siSNF2H_High-Mnase_1-3
Figure7	A	siSNF2H_RNAseq_rep1-4	siScr_RNAseq_rep1-4
	B,C	siScr_High-Mnase_1-3	siSNF2H_High-Mnase_1-3
S1	A	siScr_rep1	siCHD1_rep1
	B	siScr_rep1	siCHD2_rep2
	C	siScr_rep1	siCHD4_rep3
S2	A	siScr_rep1	siCHD1_rep1
	B	siScr_rep1	siCHD2_rep2
	C	siScr_rep1	siCHD4_rep3
S3	B	siScr_rep1	siACF1_rep2
	C	siScr_rep1	siRSF1_rep2
	D	siScr_rep1	siWSTF_rep2
	E	siScr_rep1	siTIP5_rep2
	F	siScr_rep1	siBPTF_rep2
S4	CTCF_chip	siScr_CTCF_rep2	
	Mnase	siScr_High-Mnase_1-3	
S5	SNF2H, BPTF chip	siScr_SNF2H_rep2	siScr_BPTF_rep1
	RAD21 chip	siScr_RAD21_rep1	
S6	SNF2L_Low Mnase	siScr_Low-Mnase_1 - 3	siSNF2L_Low-Mnase_1 -3

SNF2L_HighMnase	siScr_High-Mnase_1-3	siSNF2L_High-Mnase_1-2
SNF2H_Low Mnase	siScr_Low-Mnase_1 - 3	siSNF2H_Low-Mnase_1-3
SNF2H_HighMnase	siScr_High-Mnase_1-3	siSNF2H_High-Mnase_1-3
